# Supplementary material for: Discovery and application of insertion-deletion (INDEL) polymorphisms for QTL mapping of early life-history traits in Atlantic salmon
Source: BMC Genomics. 2010 Mar 8;11:156. doi: 10.1186/1471-2164-11-156 (PMC2838853; doi:10.1186/1471-2164-11-156)
Supplement: Additional file 2 — Information on developed 76 locus single-run INDEL panel in Atlantic salmon. Information on fluorescence labeling, primer concentrations, PCR pooling and links to alignments, INDEL motifs and GENESCAN (Burge and Karlin 1997) predictions of genes/exons are available in html format. [file 1471-2164-11-156-S2.ZIP › Additionalfile2/table548.html]

Contig Alignment

```
Base	A B C D E F G H I J K L M N O P Q R S T U V W X Y Z a b c d e f g   
1	. . G . . . . . . . . . . . . . . . . . . . . . . . . . . . . . .    
2	. . G . . . . . . . . . . . . . . . . . . . . . . . . . . . . . .    
3	. . G . . . . . . . . . . . . . . . . . . . . . . . . . . . . . .    
4	. . G . . . . . . . . . . . . . . . . . . . . . . . . . . . . . .    
5	. . G . . . . . . . . . . . . . . . . . . . . . . . . . . . . . .    
6	. . C . . . . . . . . . . . . . . . . . . . . . . . . . . . . . .    
7	. . C . . . . . . . . . . . . . . . . . . . . . . . . . . . . . .    
8	. . A . . . . . . . . . . . . . . . . . . . . . . . . . . . . . .    
9	. . T . . . . . . . . . . . . . . . . . . . . . . . . . . . . . .    
10	. . T . . . . . . . . . . . . . . . . . . . . . . . . . . . . . .    
11	. . T . . . . . . . . . . . . . . . . . . . . . . . . . . . . . .    
12	. . A . . . . . . . . . . . . . . . . . . . . . . . . . . . . . .    
13	. . T . . . . . . . . . . . . . . . . . . . . . . . . . . . . . .    
14	. . G . . . . . . . . . . . . . . . . . . . . . . . . . . . . . .    
15	. . G . . . . . . . . . . . . . . . . . . . . . . . . . . . . . .    
16	. . T . . . . . . . . . . . . . . . . . . . . . . . . . . . . . .    
17	. . G . . . . . . . . . . . . . . . . . . . . . . . . . . . . . .    
18	. . G . . . . . . . . . . . . . . . . . . . . . . . . . . . . . .    
19	. . G . . . . . . . . . . . . . . . . . . . . . . . . . . . . . .    
20	. . A . . . . . . . . . . . . . . . . . . . . . . . . . . . . . .    
21	. . G . . . . . . . . . . . . . . . . . . . . . . . . . . . . . .    
22	. . A . . . . . . . . . . . . . . . . . . . . . . . . . . . . . .    
23	. . G . . . . . . . . . . . . . . . . . . . . . . . . . . . . . .    
24	. . G . . . . . . . . . . . . . . . . . . . . . . . . . . . . . .    
25	. . T . . . . . . . . . . . . . . . . . . . . . . . . . . . . . .    
26	. . T . . . . . . . . . . . . . . . . . . . . . . . . . . . . . .    
27	. . A . . . . . . . . . . . . . . . . . . . . . . . . . . . . . .    
28	. . G . . . . . . . . . . . . . . . . . . . . . . . . . . . . . .    
29	. . C . . . . . . . . . . . . . . . . . . . . . . . . . . . . . .    
30	. . T . . . . . . . . . . . . . . . . . . . . . . . . . . . . . .    
31	. . A . . . . . . . . . . . . . . . . . . . . . . . . . . . . . .    
32	. . G . . . . . . . . . . . . . . . . . . . . . . . . . . . . . .    
33	. . C . . . . . . . . . . . . . . . . . . . . . . . . . . . . . .    
34	. . T . . . . . . . . . . . . . . . . . . . . . . . . . . . . . .    
35	. . A . . . . . . . . . . . . . . . . . . . . . . . . . . . . . .    
36	. . G . . . . . . . . . . . . . . . . . . . . . . . . . . . . . .    
37	. . G . . . . . . . . . . . . . . . . . . . . . . . . . . . . . .    
38	. . G . . . . . . . . . . . . . . . . . . . . . . . . . . . . . .    
39	. . A . . . . . . . . . . . . . . . . . . . . . . . . . . . . . .    
40	. . T . . . . . . . . . . . . . . . . . . . . . . . . . . . . . .    
41	. . T . . . . . . . . . . . . . . . . . . . . . . . . . . . . . .    
42	. . G . . . . . . . . . . . . . . . . . . . . . . . . . . . . . .    
43	. . A . . . . . . . . . . . . . . . . . . . . . . . . . . . . . .    
44	. . C . . . . . . . . . . . . . . . . . . . . . . . . . . . . . .    
45	. . G . . . . . . . . . . . . . . . . . . . . . . . . . . . . . .    
46	. . T . . . . . . . . . . . . . . . . . . . . . . . . . . . . . .    
47	. . T . . . . . . . . . . . . . . . . . . . . . . . . . . . . . .    
48	. . C . . . . . . . . . . . . . . . . . . . . . . . . . . . . . .    
49	. . G . . . . . . . . . . . . . . . . . . . . . . . . . . . . . .    
50	. . C C . . . . . . . . . . . . . . . . . . . . . . . . . . . . .    
51	. . G G . G . . . . . . . . . . . . . . . . . . . . . . . . . . .    
52	. . G G . G . . . . . . . . . . . . . . . . . . . . . . . . . . .    
53	. . A A . A . . . . . . . . . . . . . . . . . . . . . . . . . . .    
54	. . G G . G . . . . . . . . . . . . . . . . . . . . . . . . . . .    
55	. . G G . G . . . . . . . . . . . . . . . . . . . . . . . . . . .    
56	. . T T . T . . . . . . . . . . . . . . . . . . . . . . . . . . .    
57	. . T T . T . . . . . . . . . . . . . . . . . . . . . . . . . . .    
58	. . C C . C . . . . . . . . . . . . . . . . . . . . . . . . . . .    
59	. . A A . A . . . . . . . . . . . . . . . . . . . . . . . . . . .    
60	. . T T . T . . . . . . . . . . . . . . . . . . . . . . . . . . .    
61	. . T T . T . . . . . . . . . . . . . . . . . . . . . . . . . . .    
62	. G G G G G . . . . . . . . . . . . . . . . . . . . . . . . . . .    
63	. A A A A A . . . . . . . . . . . . . . . . . . . . . . . . . . .    
64	. A A A A A . . . . . . . . . . . . . . . . . . . . . . . . . . .    
65	. G G G G G . . . . . . . . . . . . . . . . . . . . . . . . . . .    
66	. T T T T T . . . . . . . . . . . . . . . . . . . . . . . . . . .    
67	. C C C C C . . . . . . . . . . . . . . . . . . . . . . . . . . .    
68	. A A A A A . . . . . . . . . . . . . . . . . . . . . . . . . . .    
69	. G G G G G . . . . . . . . . . . . . . . . . . . . . . . . . . .    
70	. C C C C C . . . . . . . . . . . . . . . . . . . . . . . . . . .    
71	. T T T T T . . . . . . . . . . . . . . . . . . . . . . . . . . .    
72	. A A A A A . . . . . . . . . . . . . . . . . . . . . . . . . . .    
73	. C C C C C . . . . . . . . . . . . . . . . . . . . . . . . . . .    
74	. T T T T T . . . . . . . . . . . . . . . . . . . . . . . . . . .    
75	. C C C C C . . . . . . . . . . . . . . . . . . . . . . . . . . .    
76	. C C C C C . . . . . . . . . . . . . . . . . . . . . . . . . . .    
77	. A A A A A . . . . . . . . . . . . . . . . . . . . . . . . . . .    
78	. G G G G G . . . . . . . . . . . . . . . . . . . . . . . . . . .    
79	. T T T T T . . . . . . . . . . . . . . . . . . . . . . . . . . .    
80	. G G G G G . . . . . . . . . . . . . . . . . . . . . . . . . . .    
81	. A A A A A . . . . . . . . . . . . . . . . . . . . . . . . . . .    
82	. G G G G G . . . . . . . . . . . . . . . . . . . . . . . . . . .    
83	. G G G G G . . . . . . . . . . . . . . . . . . . . . . . . . . .    
84	. T T T T T . . . . . . . . . . . . . . . . . . . . . . . . . . .    
85	. G G G G G . . . . . . . . . . . . . . . . . . . . . . . . . . .    
86	. G G G G G . . . . . . . . . . . . . . . . . . . . . . . . . . .    
87	. C C C C C . . . . . . . . . . . . . . . . . . . . . . . . . . .    
88	. G G G G G . . . . . . . . . . . . . . . . . . . . . . . . . . .    
89	. G G G G G . . . . . . . . . . . . . . . . . . . . . . . . . . .    
90	. G G G G G . . . . . . . . . . . . . . . . . . . . . . . . . . .    
91	. G G G G G . . . . . . . . . . . . . . . . . . . . . . . . . . .    
92	. A A A A A . . . . . . . . . . . . . . . . . . . . . . . . . . .    
93	. G G G G G . . . . . . . . . . . . . . . . . . . . . . . . . . .    
94	. A A A A A . . . . . . . . . . . . . . . . . . . . . . . . . . .    
95	. C C C C C . . . . . . . . . . . . . . . . . . . . . . . . . . .    
96	. A A A A A . . . . . . . . . . . . . . . . . . . . . . . . . . .    
97	. A A A A A . . . . . . . . . . . . . . . . . . . . . . . . . . .    
98	. A A A A A . . . . . . . . . . . . . . . . . . . . . . . . . . .    
99	. A A A A A . . . . . . . . . . . . . . . . . . . . . . . . . . .    
100	. C C C C C . . . . . . . . . . . . . . . . . . . . . . . . . . .    
101	. A A A A A . . . . . . . . . . . . . . . . . . . . . . . . . . .    
102	. C C C C C . . . . . . . . . . . . . . . . . . . . . . . . . . .    
103	. G G G G G . . . . . . . . . . . . . . . . . . . . . . . . . . .    
104	. A A A A A . . . . . . . . . . . . . . . . . . . . . . . . . . .    
105	C C C C C C . . . . . . . . . . . . . . . . . . . . . . . . . . .    
106	T T T T T T . . . . . . . . . . . . . . . . . . . . . . . . . . .    
107	T T T T T T . . . . . . . . . . . . . . . . . . . . . . . . . . .    
108	T T T T T T . . . . . . . . . . . . . . . . . . . . . . . . . . .    
109	T T T T T T . . . . . . . . . . . . . . . . . . . . . . . . . . .    
110	G G G G G G . . . . . . . . . . . . . . . . . . . . . . . . . . .    
111	T T T T T T . . . . . . . . . . . . . . . . . . . . . . . . . . .    
112	G G G G G G . . . . . . . . . . . . . . . . . . . . . . . . . . .    
113	C C C C C C . . . . . . . . . . . . . . . . . . . . . . . . . . .    
114	C C C C C C . . . . . . . . . . . . . . . . . . . . . . . . . . .    
115	G G G G G G . . . . . . . . . . . . . . . . . . . . . . . . . . .    
116	T T T T T T . . . . . . . . . . . . . . . . . . . . . . . . . . .    
117	C C C C C C . . . . . . . . . . . . . . . . . . . . . . . . . . .    
118	G G G G G G . . . . . . . . . . . . . . . . . . . . . . . . . . .    
119	A A A A A A . . . . . . . . . . . . . . . . . . . . . . . . . . .    
120	G G G G G G . . . . . . . . . . . . . . . . . . . . . . . . . . .    
121	T T T T T T . . . . . . . . . . . . . . . . . . . . . . . . . . .    
122	G G G G G G . . . . . . . . . . . . . . . . . . . . . . . . . . .    
123	A A A A A A . . . . . . . . . . . . . . . . . . . . . . . . . . .    
124	A A A A A A . . . . . . . . . . . . . . . . . . . . . . . . . . .    
125	G G G G G G . . . . . . . . . . . . . . . . . . . . . . . . . . .    
126	T T T T T T . . . . . . . . . . . . . . . . . . . . . . . . . . .    
127	A A A A A A . . . . . . . . . . . . . . . . . . . . . . . . . . .    
128	A A A A A A . . . . . . . . . . . . . . . . . . . . . . . . . . .    
129	C C C C C C . . . . . . . . . . . . . . . . . . . . . . . . . . .    
130	C C C C C C . . . . . . . . . . . . . . . . . . . . . . . . . . .    
131	C C C C C C . . . . . . . . . . . . . . . . . . . . . . . . . . .    
132	C C C C C C . . . . . . . . . . . . . . . . . . . . . . . . . . .    
133	A A A A A A . . . . . . . . . . . . . . . . . . . . . . . . . . .    
134	C C C C C C . . . . . . . . . . . . . . . . . . . . . . . . . . .    
135	C C C C C C . . . . . . . . . . . . . . . . . . . . . . . . . . .    
136	A A A A A A . . . . . . . . . . . . . . . . . . . . . . . . . . .    
137	T T T T T T . . . . . . . . . . . . . . . . . . . . . . . . . . .    
138	G G G G G G . . . . . . . . . . . . . . . . . . . . . . . . . . .    
139	C C C C C C . . . . . . . . . . . . . . . . . . . . . . . . . . .    
140	G G G G G G . . . . . . . . . . . . . . . . . . . . . . . . . . .    
141	T T T T T T . . . . . . . . . . . . . . . . . . . . . . . . . . .    
142	G G G G G G . . . . . . . . . . . . . . . . . . . . . . . . . . .    
143	A A A A A A . . . . . . . . . . . . . . . . . . . . . . . . . . .    
144	A A A A A A . . . . . . . . . . . . . . . . . . . . . . . . . . .    
145	T T T T T T . . . . . . . . . . . . . . . . . . . . . . . . . . .    
146	A A A A A A . . . . . . . . . . . . . . . . . . . . . . . . . . .    
147	C C C C C C . . . . . . . . . . . . . . . . . . . . . . . . . . .    
148	A A A A A A . . . . . . . . . . . . . . . . . . . . . . . . . . .    
149	A A A A A A . . . . . . . . . . . . . . . . . . . . . . . . . . .    
150	G G G G G G . . . . . . . . . . . . . . . . . . . . . . . . . . .    
151	C C C C C C . . . . . . . . . . . . . . . . . . . . . . . . . . .    
152	T T T T T T . . . . . . . . . . . . . . . . . . . . . . . . . . .    
153	A A A A A A . . . . . . . . . . . . . . . . . . . . . . . . . . .    
154	G G G G G G . . . . . . . . . . . . . . . . . . . . . . . . . . .    
155	T T T T T T . . . . . . . . . . . . . . . . . . . . . . . . . . .    
156	G G G G G G . . . . . . . . . . . . . . . . . . . . . . . . . . .    
157	G G G G G G . . . . . . . . . . . . . . . . . . . . . . . . . . .    
158	T T T T T T . . . . . . . . . . . . . . . . . . . . . . . . . . .    
159	G G G G G G . . . . . . . . . . . . . . . . . . . . . . . . . . .    
160	T T T T T T . . . . . . . . . . . . . . . . . . . . . . . . . . .    
161	T T T T T T . . . . . . . . . . . . . . . . . . . . . . . . . . .    
162	A A A A A A . . . . . . . . . . . . . . . . . . . . . . . . . . .    
163	G G G G G G . . . . . . . . . . . . . . . . . . . . . . . . . . .    
164	G G G G G G . . . . . . . . . . . . . . . . . . . . . . . . . . .    
165	C C C C C C . . . . . . . . . . . . . . . . . . . . . . . . . . .    
166	T T T T T T . . . . . . . . . . . . . . . . . . . . . . . . . . .    
167	C C C C C C . . . . . . . . . . . . . . . . . . . . . . . . . . .    
168	A A A A A A . . . . . . . . . . . . . . . . . . . . . . . . . . .    
169	G G G G G G . . . . . . . . . . . . . . . . . . . . . . . . . . .    
170	G G G G G G . . . . . . . . . . . . . . . . . . . . . . . . . . .    
171	A A A A A A . . . . . . . . . . . . . . . . . . . . . . . . . . .    
172	G G G G G G . . . . . . . . . . . . . . . . . . . . . . . . . . .    
173	G G G G G G . . . . . . . . . . . . . . . . . . . . . . . . . . .    
174	T T T T T T . . . . . . . . . . . . . . . . . . . . . . . . . . .    
175	G G G G G G . . . . . . . . . . . . . . . . . . . . . . . . . . .    
176	T T T T T T . . . . . . . . . . . . . . . . . . . . . . . . . . .    
177	A A A A A A . . . . . . . . . . . . . . . . . . . . . . . . . . .    
178	G G G G G G . . . . . . . . . . . . . . . . . . . . . . . . . . .    
179	G G G G G G . . . . . . . . . . . . . . . . . . . . . . . . . . .    
180	C C C C C C . . . . . . . . . . . . . . . . . . . . . . . . . . .    
181	A A A A A A . . . . . . . . . . . . . . . . . . . . . . . . . . .    
182	A A A A A A . . . . . . . . . . . . . . . . . . . . . . . . . . .    
183	A A A A A A . . . . . . . . . . . . . . . . . . . . . . . . . . .    
184	T T T T T T . . . . . . . . . . . . . . . . . . . . . . . . . . .    
185	C C C C C C . . . . . . . . . . . . . . . . . . . . . . . . . . .    
186	T T T T T T . . . . . . . . . . . . . . . . . . . . . . . . . . .    
187	G G G G G G . . . . . . . . . . . . . . . . . . . . . . . . . . .    
188	C C C C C C . . . . . . . . . . . . . . . . . . . . . . . . . . .    
189	A A A A A A . . . . . . . . . . . . . . . . . . . . . . . . . . .    
190	C C C C C C . . . . . . . . . . . . . . . . . . . . . . . . . . .    
191	T T T T T T . . . . . . . . . . . . . . . . . . . . . . . . . . .    
192	G G G G G G . . . . . . . . . . . . . . . . . . . . . . . . . . .    
193	A A A A A A . . . . . . . . . . . . . . . . . . . . . . . . . . .    
194	C C C C C C . . . . . . . . . . . . . . . . . . . . . . . . . . .    
195	T T T T T T . . . . . . . . . . . . . . . . . . . . . . . . . . .    
196	G G G G G G . . . . . . . . . . . . . . . . . . . . . . . . . . .    
197	T T T T T T . . . . . . . . . . . . . . . . . . . . . . . . . . .    
198	C C C C C C . . . . . . . . . . . . . . . . . . . . . . . . . . .    
199	C C C C C C . . . . . . . . . . . . . . . . . . . . . . . . . . .    
200	A A A A A A . . . . . . . . . . . . . . . . . . . . . . . . . . .    
201	A A A A A A . . . . . . . . . . . . . . . . . . . . . . . . . . .    
202	T T T T T T . . . . . . . . . . . . . . . . . . . . . . . . . . .    
203	T T T T T T . . . . . . . . . . . . . . . . . . . . . . . . . . .    
204	T T T T T T . . . . . . . . . . . . . . . . . . . . . . . . . . .    
205	G G G G G G . . . . . . . . . . . . . . . . . . . . . . . . . . .    
206	T T T T T T . . . . . . . . . . . . . . . . . . . . . . . . . . .    
207	A A A A A A . . . . . . . . . . . . . . . . . . . . . . . . . . .    
208	C C C C C C . . . . . . . . . . . . . . . . . . . . . . . . . . .    
209	A A A A A A . . . . . . . . . . . . . . . . . . . . . . . . . . .    
210	G G G G G G . . . . . . . . . . . . . . . . . . . . . . . . . . .    
211	G G G G G G . . . . . . . . . . . . . . . . . . . . . . . . . . .    
212	G G G G G G . . . . . . . . . . . . . . . . . . . . . . . . . . .    
213	T T T T T T . . . . . . . . . . . . . . . . . . . . . . . . . . .    
214	A A A A A A . . . . . . . . . . . . . . . . . . . . . . . . . . .    
215	T T T T T T . . . . . . . . . . . . . . . . . . . . . . . . . . .    
216	T T T T T T . . . . . . . . . . . . . . . . . . . . . . . . . . .    
217	T T T T T T . . . . . . . . . . . . . . . . . . . . . . . . . . .    
218	T T T T T T . . . . . . . . . . . . . . . . . . . . . . . . . . .    
219	T T T T T T . . . . . . . . . . . . . . . . . . . . . . . . . . .    
220	G G G G G G . . . . . . . . . . . . . . . . . . . . . . . . . . .    
221	T T T T T T . . . . . . . . . . . . . . . . . . . . . . . . . . .    
222	G G G G G G . . . . . . . . . . . . . . . . . . . . . . . . . . .    
223	G G G G G G . . . . . . . . . . . . . . . . . . . . . . . . . . .    
224	A A A A A A . . . . . . . . . . . . . . . . . . . . . . . . . . .    
225	A A A A A A . . . . . . . . . . . . . . . . . . . . . . . . . . .    
226	A A A A A A . . . . . . . . . . . . . . . . . . . . . . . . . . .    
227	A A A A A A . . . . . . . . . . . . . . . . . . . . . . . . . . .    
228	A A A A A A . . . . . . . . . . . . . . . . . . . . . . . . . . .    
229	T T T T T T . . . . . . . . . . . . . . . . . . . . . . . . . . .    
230	A A A A A A . . . . . . . . . . . . . . . . . . . . . . . . . . .    
231	T T T T T T . . . . . . . . . . . . . . . . . . . . . . . . . . .    
232	G G G G G G . . . . . . . . . . . . . . . . . . . . . . . . . . .    
233	A A A A A A . . . . . . . . . . . . . . . . . . . . . . . . . . .    
234	C C C C C C . . . . . . . . . . . . . . . . . . . . . . . . . . .    
235	C C C C C C . . . . . . . . . . . . . . . . . . . . . . . . . . .    
236	C C C C C C . . . . . . . . . . . . . . . . . . . . . . . . . . .    
237	C C C C C C . . . . . . . . . . . . . . . . . . . . . . . . . . .    
238	A A A A A A . . . . . . . . . . . . . . . . . . . . . . . . . . .    
239	C C C C C C . . . . . . . . . . . . . . . . . . . . . . . . . . .    
240	A A A A A A . . . . . . . . . . . . . . . . . . . . . . . . . . .    
241	A A A A A A . . . . . . . . . . . . . . . . . . . . . . . . . . .    
242	T T T T T T . . . . . . . . . . . . . . . . . . . . . . . . . . .    
243	A A A A A A . . . . . . . . . . . . . . . . . . . . . . . . . . .    
244	G G G G G G . . . . . . . . . . . . . . . . . . . . . . . . . . .    
245	A A A A A A . . . . . . . . . . . . . . . . . . . . . . . . . . .    
246	A A A A A A . . . . . . . . . . . . . . . . . . . . . . . . . . .    
247	G G G G G G . . . . . . . . . . . . . . . . . . . . . . . . . . .    
248	A A A A A A . . . . . . . . . . . . . . . . . . . . . . . . . . .    
249	C C C C C C . . . . . . . . . . . . . . . . . . . . . . . . . . .    
250	T T T T T T . . . . . . . . . . . . . . . . . . . . . . . . . . .    
251	C C C C C C . . . . . . . . . . . . . . . . . . . . . . . . . . .    
252	C C C C C C . . . . . . . . . . . . . . . . . . . . . . . . . . .    
253	T T T T T T . . . . . . . . . . . . . . . . . . . . . . . . . . .    
254	A A A A A A . . . . . . . . . . . . . . . . . . . . . . . . . . .    
255	C C C C C C . . . . . . . . . . . . . . . . . . . . . . . . . . .    
256	A A A A A A . . . . . . . . . . . . . . . . . . . . . . . . . . .    
257	G G G G G G . . . . . . . . . . . . . . . . . . . . . . . . . . .    
258	A A A A A A . . . . . . . . . . . . . . . . . . . . . . . . . . .    
259	A A A A A A . . . . . . . . . . . . . . . . . . . . . . . . . . .    
260	A A A A A A . . . . . . . . . . . . . . . . . . . . . . . . . . .    
261	G G G G G G . . . . . . . . . . . . . . . . . . . . . . . . . . .    
262	C C C C C C . . . . . . . . . . . . . . . . . . . . . . . . . . .    
263	A A A A A A . . . . . . . . . . . . . . . . . . . . . . . . . . .    
264	A A A A A A . . . . . . . . . . . . . . . . . . . . . . . . . . .    
265	G G G G G G . . . . . . . . . . . . . . . . . . . . . . . . . . .    
266	T T T T T T . . . . . . . . . . . . . . . . . . . . . . . . . . .    
267	T T T T T T . . . . . . . . . . . . . . . . . . . . . . . . . . .    
268	G G G G A G . . . . . . . . . . . . . . . . . . . . . . . . . . .    
269	A A A A A A . . . . . . . . . . . . . . . . . . . . . . . . . . .    
270	A A A A A A . . . . . . . . . . . . . . . . . . . . . . . . . . .    
271	G G G G G G . . . . . . . . . . . . . . . . . . . . . . . . . . .    
272	T T T T T T . . . . . . . . . . . . . . . . . . . . . . . . . . .    
273	G G G G G G . . . . . . . . . . . . . . . . . . . . . . . . . . .    
274	G G G G G G . . . . . . . . . . . . . . . . . . . . . . . . . . .    
275	. A A A A A . . . . . . . . . . . . . . . . . . . . . . . . . . .    
276	. T T T T T . . . . . . . . . . . . . . . . . . . . . . . . . . .    
277	. G G G G G . . . . . . . . . . . . . . . . . . . . . . . . . . .    
278	. G G G G G . . . . . . . . . . . . . . . . . . . . . . . . . . .    
279	. G G G G G . . . . . . . . . . . . . . . . . . . . . . . . . . .    
280	. C C C C C . . . . . . . . . . . . . . . . . . . . . . . . . . .    
281	. A A A A A . . . . . . . . . . . . . . . . . . . . . . . . . . .    
282	. G G G G G . . . . . . . . . . . . . . . . . . . . . . . . . . .    
283	. C C C C C . . . . . . . . . . . . . . . . . . . . . . . . . . .    
284	. A A A A A . . . . . . . . . . . . . . . . . . . . . . . . . . .    
285	. G G G G G . . . . . . . . . . . . . . . . . . . . . . . . . . .    
286	. T T T T T . . . . . . . . . . . . . . . . . . . . . . . . . . .    
287	. G G G G G . . . . . . . . . . . . . . . . . . . . . . . . . . .    
288	. C C C C C . . . . . . . . . . . . . . . . . . . . . . . . . . .    
289	. A A A A A . . . . . . . . . . . . . . . . . . . . . . . . . . .    
290	. T T T T T . . . . . . . . . . . . . . . . . . . . . . . . . . .    
291	. G G G G G . . . . . . . . . . . . . . . . . . . . . . . . . . .    
292	. C C C C C . . . . . . . . . . . . . . . . . . . . . . . . . . .    
293	. T T T T T . . . . . . . . . . . . . . . . . . . . . . . . . . .    
294	. T T T T T . . . . . . . . . . . . . . . . . . . . . . . . . . .    
295	. G G G G G . . . . . . . . . . . . . . . . . . . . . . . . . . .    
296	. A A A A A . . . . . . . . . . . . . . . . . . . . . . . . . . .    
297	. A A A A A . . . . . . . . . . . . . . . . . . . . . . . . . . .    
298	. A A A A A . . . . . . . . . . . . . . . . . . . . . . . . . . .    
299	. T T T T T . . . . . . . . . . . . . . . . . . . . . . . . . . .    
300	. C C C C C . . . . . . . . . . . . . . . . . . . . . . . . . . .    
301	. C C C C C . . . . . . . . . . . . . . . . . . . . . . . . . . .    
302	. T T T T T . . . . . . . . . . . . . . . . . . . . . . . . . . .    
303	. T T T T T . . . . . . . . . . . . . . . . . . . . . . . . . . .    
304	. G G G G G . . . . . . . . . . . . . . . . . . . . . . . . . . .    
305	. A A A A A . . . . . . . . . . . . . . . . . . . . . . . . . . .    
306	. C C C C C . . . . . . . . . . . . . . . . . . . . . . . . . . .    
307	. A A A A A . . . . . . . . . . . . . . . . . . . . . . . . . . .    
308	. C C C C C . . . . . . . . . . . . . . . . . . . . . . . . . . .    
309	. A A A G A . . . . . . . . . . . . . . . . . . . . . . . . . . .    
310	. G G G G G . . . . . . . . . . . . . . . . . . . . . . . . . . .    
311	. C C C C C . . . . . . . . . . . . . . . . . . . . . . . . . . .    
312	. T T T T T . . . . . . . . . . . . . . . . . . . . . . . . . . .    
313	. G G G G G . . . . . . . . . . . . . . . . . . . . . . . . . . .    
314	. G G G G G . . . . . . . . . . . . . . . . . . . . . . . . . . .    
315	. A A A A A . . . . . . . . . . . . . . . . . . . . . . . . . . .    
316	. A A A A A . . . . . . . . . . . . . . . . . . . . . . . . . . .    
317	. C C C C C . . . . . . . . . . . . . . . . . . . . . . . . . . .    
318	. A A A A A . . . . . . . . . . . . . . . . . . . . . . . . . . .    
319	. G G G G G . . . . . . . . . . . . . . . . . . . . . . . . . . .    
320	. A A A A A . . . . . . . . . . . . . . . . . . . . . . . . . . .    
321	. G G G G G . . . . . . . . . . . . . . . . . . . . . . . . . . .    
322	. C C C C C . . . . . . . . . . . . . . . . . . . . . . . . . . .    
323	. A A A A A . . . . . . . . . . . . . . . . . . . . . . . . . . .    
324	. G G G G G . . . . . . . . . . . . . . . . . . . . . . . . . . .    
325	. T T T T T . . . . . . . . . . . . . . . . . . . . . . . . . . .    
326	. T T T T T . . . . . . . . . . . . . . . . . . . . . . . . . . .    
327	. C C C C C . . . . . . . . . . . . . . . . . . . . . . . . . . .    
328	. A A A A A . . . . . . . . . . . . . . . . . . . . . . . . . . .    
329	. C C C C C . . . . . . . . . . . . . . . . . . . . . . . . . . .    
330	. G G G G G . . . . . . . . . . . . . . . . . . . . . . . . . . .    
331	. G G G G G . . . . . . . . . . . . . . . . . . . . . . . . . . .    
332	. C C C C C . . . . . . . . . . . . . . . . . . . . . . . . . . .    
333	. G G G G G . . . . . . . . . . . . . . . . . . . . . . . . . . .    
334	. A A A A A . . . . . . . . . . . . . . . . . . . . . . . . . . .    
335	. T T T T T . . . . . . . . . . . . . . . . . . . . . . . . . . .    
336	. G G G G G . . . . . . . . . . . . . . . . . . . . . . . . . . .    
337	. A A A A A . . . . . . . . . . . . . . . . . . . . . . . . . . .    
338	. G G G G G . . . . . . . . . . . . . . . . . . . . . . . . . . .    
339	. G G G G G . . . . . . . . . . . . . . . . . . . . . . . . . . .    
340	. G G G G G . . . . . . . . . . . . . . . . . . . . . . . . . . .    
341	. A A A A A . . . . . . . . . . . . . . . . . . . . . . . . . . .    
342	. C C C C C . . . . . . . . . . . . . . . . . . . . . . . . . . .    
343	. T T T T T . . . . . . . . . . . . . . . . . . . . . . . . . . .    
344	. T T T T T . . . . . . . . . . . . . . . . . . . . . . . . . . .    
345	. G G G G G . . . . . . . . . . . . . . . . . . . . . . . . . . .    
346	. T T T T T . . . . . . . . . . . . . . . . . . . . . . . . . . .    
347	. A A A A A . . . . . . . . . . . . . . . . . . . . . . . . . . .    
348	. C C C C C . . . . . . . . . . . . . . . . . . . . . . . . . . .    
349	. A A A A A . . . . . . . . . . . . . . . . . . . . . . . . . . .    
350	. T T T T T . . . . . . . . . . . . . . . . . . . . . . . . . . .    
351	. G G G G G . . . . . . . . . . . . . . . . . . . . . . . . . . .    
352	. A A A A A . . . . . . . . . . . . . . . . . . . . . . . . . . .    
353	. A A A A A . . . . . . . . . . . . . . . . . . . . . . . . . . .    
354	. G G G G G . . . . . . . . . . . . . . . . . . . . . . . . . . .    
355	. A A A A A . . . . . . . . . . . . . . . . . . . . . . . . . . .    
356	. A A A A A . . . . . . . . . . . . . . . . . . . . . . . . . . .    
357	. C C C C C . . . . . . . . . . . . . . . . . . . . . . . . . . .    
358	. G G G G G . . . . . . . . . . . . . . . . . . . . . . . . . . .    
359	. G G G G G . . . . . . . . . . . . . . . . . . . . . . . . . . .    
360	. T T T T T . . . . . . . . . . . . . . . . . . . . . . . . . . .    
361	. C C C C C . . . . . . . . . . . . . . . . . . . . . . . . . . .    
362	. A A A A A . . . . . . . . . . . . . . . . . . . . . . . . . . .    
363	. A A A A A . . . . . . . . . . . . . . . . . . . . . . . . . . .    
364	. G G G G G . . . . . . . . . . . . . . . . . . . . . . . . . . .    
365	. G G G G G . . . . . . . . . . . . . . . . . . . . . . . . . . .    
366	. C C C C C . . . . . . . . . . . . . . . . . . . . . . . . . . .    
367	. T T T T T . . . . . . . . . . . . . . . . . . . . . . . . . . .    
368	. T T T T T . . . . . . . . . . . . . . . . . . . . . . . . . . .    
369	. C T C C C . . . . . . . . . . . . . . . . . . . . . . . . . . .    
370	. G G G G G . . . . . . . . . . . . . . . . . . . . . . . . . . .    
371	. C C C C C . . . . . . . . . . . . . . . . . . . . . . . . . . .    
372	. T T T T T . . . . . . . . . . . . . . . . . . . . . . . . . . .    
373	. C C C C C . . . . . . . . . . . . . . . . . . . . . . . . . . .    
374	. T T T T T . . . . . . . . . . . . . . . . . . . . . . . . . . .    
375	. T T T T T . . . . . . . . . . . . . . . . . . . . . . . . . . .    
376	. G G G G G . . . . . . . . . . . . . . . . . . . . . . . . . . .    
377	. T T T T T . . . . . . . . . . . . . . . . . . . . . . . . . . .    
378	. A A A A A . . . . . . . . . . . . . . . . . . . . . . . . . . .    
379	. T T T T T . . . . . . . . . . . . . . . . . . . . . . . . . . .    
380	. A A A A A . . . . . . . . . . . . . . . . . . . . . . . . . . .    
381	. C C C C C . . . . . . . . . . . . . . . . . . . . . . . . . . .    
382	. T T T T T . . . . . . . . . . . . . . . . . . . . . . . . . . .    
383	. C C C C C . . . . . . . . . . . . . . . . . . . . . . . . . . .    
384	. C C C C C . . . . . . . . . . . . . . . . . . . . . . . . . . .    
385	. A A A A G . . . . . . . . . . . . . . . . . . . . . . . . . . .    
386	. T T T T T . . . . . . . . . . . . . . . . . . . . . . . . . . .    
387	. C C C C C . . . . . . . . . . . . . . . . . . . . . . . . . . .    
388	. A A A A A . . . . . . . . . . . . . . . . . . . . . . . . . . .    
389	. C C C C C . . . . . . . . . . . . . . . . . . . . . . . . . . .    
390	. G G G G G . . . . . . . . . . . . . . . . . . . . . . . . . . .    
391	. G G G G G . . . . . . . . . . . . . . . . . . . . . . . . . . .    
392	. C C C C C . . . . . . . . . . . . . . . . . . . . . . . . . . .    
393	. A A A A A . . . . . . . . . . . . . . . . . . . . . . . . . . .    
394	. C C C C C . . . . . . . . . . . . . . . . . . . . . . . . . . .    
395	. A A A A A . . . . . . . . . . . . . . . . . . . . . . . . . . .    
396	. G G G G G . . . . . . . . . . . . . . . . . . . . . . . . . . .    
397	. T T T T T . . . . . . . . . . . . . . . . . . . . . . . . . . .    
398	. C C C C C . . . . . . . . . . . . . . . . . . . . . . . . . . .    
399	. C C C C C . . . . . . . . . . . . . . . . . . . . . . . . . . .    
400	. A A A A A . . . . . . . . . . . . . . . . . . . . . . . . . . .    
401	. C C C C C . . . . . . . . . . . . . . . . . . . . . . . . . . .    
402	. G G G G G . . . . . . . . . . . . . . . . . . . . . . . . . . .    
403	. T T T T T . . . . . . . . . . . . . . . . . . . . . . . . . . .    
404	. T T T T T . . . . . . . . . . . . . . . . . . . . . . . . . . .    
405	. T T T T T . T . . . . . . . . . . . . . . . . . . . . . . . . .    
406	. A A A A A . A . A . . . . . . . . . . . . . . . . . . . . . . .    
407	. A A A A A . A . A . . . . . . . . . . . . . . . . . . . . . . .    
408	. T T T T T . T . T . . . . . . . . . . . . . . . . . . . . . . .    
409	. G G G G G . G . G . . . . . . . . . . . . . . . . . . . . . . .    
410	. A A A A A . A . A . . . . . . . . . . . . . . . . . . . . . . .    
411	. T T T T T . T . T . . . . . . . . . . . . . . . . . . . . . . .    
412	. C C C C C . C . C . . . . . . . . . . . . . . . . . . . . . . .    
413	. T T T T T . T . T . . . . . . . . . . . . . . . . . . . . . . .    
414	. A A A A A . A . A . . . . . . . . . . . . . . . . . . . . . . .    
415	. C C C C C . C . C . . . . . . . . . . . . . . . . . . . . . . .    
416	. A A A A A . A . A . . . . . . . . . . . . . . . . . . . . . . .    
417	. G G G G G . G . G . . . . . . . . . . . . . . . . . . . . . . .    
418	. G G G G G . G . G . . . . . . . . . . . . . . . . . . . . . . .    
419	. A A A A A . A . A . . . . . . . . . . . . . . . . . . . . . . .    
420	. C C C C C . C . C . . . . . . . . . . . . . . . . . . . . . . .    
421	. C C C C C . C . C . . . . . . . . . . . . . . . . . . . . . . .    
422	. T T T T T . T . T . . . . . . . . . . . . . . . . . . . . . . .    
423	. T T T T T . T . T . . . . . . . . . . . . . . . . . . . . . . .    
424	. C C C C C . C . C . . . . . . . . . . . . . . . . . . . . . . .    
425	. G G G G G . G . G . . . . . . . . . . . . . . . . . . . . . . .    
426	. G G G G G . G . G . . . . . . . . . . . . . . . . . . . . . . .    
427	. G G G G G . G . G . . . . . . . . . . . . . . . . . . . . . . .    
428	. A A A A A . A . A . . . . . . . . . . . . . . . . . . . . . . .    
429	. A A A A A . A . A . . . . . . . . . . . . . . . . . . . . . . .    
430	. C C C C C . C . C . . . . . . . . . . . . . . . . . . . . . . .    
431	. A A A A A . A . A . . . . . . . . . . . . . . . . . . . . . . .    
432	. G G G G G . G . G . . . . . . . . . . . . . . . . . . . . . . .    
433	. A A A A A . A . A . . . . . . . . . . . . . . . . . . . . . . .    
434	. T T T T T . T . T . . . . . . . . . . . . . . . . . . . . . . .    
435	. C C C C C . C . C . . . . . . . . . . . . . . . . . . . . . . .    
436	. C C C C C . C . C . . . . . . . . . . . . . . . . . . . . . . .    
437	. T T T T T . T . T . . . . . . . . . . . . . . . . . . . . . . .    
438	. G G G G G . G . G . . . . . . . . . . . . . . . . . . . . . . .    
439	. A A A A A . A . A . . . . . . . . . . . . . . . . . . . . . . .    
440	. G G G G G . G . G . . . . . . . . . . . . . . . . . . . . . . .    
441	. A A A A A . A . A . . . . . . . . . . . . . . . . . . . . . . .    
442	. G G G G G . G . G . . . . . . . . . . . . . . . . . . . . . . .    
443	. T T T T T . T . T . . . . . . . . . . . . . . . . . . . . . . .    
444	. A A A A A . A . A . . . . . . . . . . . . . . . . . . . . . . .    
445	. A A A A A . A . A . . . . . . . . . . . . . . . . . . . . . . .    
446	. A A A A A . A . A . . . . . . . . . . . . . . . . . . . . . . .    
447	. G G G G G . G . G . . . . . . . . . . . . . . . . . . . . . . .    
448	. G G G G G . G . G . . . . . . . . . . . . . . . . . . . . . . .    
449	. A A A A A . A . A . . . . . . . . . . . . . . . . . . . . . . .    
450	. C C C C C . C . C . . . . . . . . . . . . . . . . . . . . . . .    
451	. A A A A A . A . A . . . . . . . . . . . . . . . . . . . . . . .    
452	. C C C C C . C . C . . . . . . . . . . . . . . . . . . . . . . .    
453	. T T T T T . T . T . . . . . . . . . . . . . . . . . . . . . . .    
454	. G G G G G . G . G . . . . . . . . . . . . . . . . . . . . . . .    
455	. A A A A A . A . A . . . . . . . . . . . . . . . . . . . . . . .    
456	. G G G G G . G . G . . . . . . . . . . . . . . . . . . . . . . .    
457	. G G G G G . G . G . . . . . . . . . . . . . . . . . . . . . . .    
458	. A A A A A . A . A . . . . . . . . . . . . . . . . . . . . . . .    
459	. C C C C C . C . C . . . . . . . . . . . . . . . . . . . . . . .    
460	. G G G G G . G . G . . . . . . . . . . . . . . . . . . . . . . .    
461	. T T T T T . T . T . . . . . . . . . . . . . . . . . . . . . . .    
462	. C C C C C . C . C . . . . . . . . . . . . . . . . . . . . . . .    
463	. C C C C C . C . C . . . . . . . . . . . . . . . . . . . . . . .    
464	. C C C C C . C . C . . . . . . . . . . . . . . . . . . . . . . .    
465	. C C C C C . C . C . . . . . . . . . . . . . . . . . . . . . . .    
466	. A A A A A . A . A . . . . . . . . . . . . . . . . . . . . . . .    
467	. T T T T T . T . T . . . . . . . . . . . . . . . . . . . . . . .    
468	. G G G G G . G . G . . . . . . . . . . . . . . . . . . . . . . .    
469	. A A A A A . A . A . . . . . . . . . . . . . . . . . . . . . . .    
470	. T T T T T . T . T . . . . . . . . . . . . . . . . . . . . . . .    
471	. C C C C C . C . C . . . . . . . . . . . . . . . . . . . . . . .    
472	. C C C C C . C . C . . . . . . . . . . . . . . . . . . . . . . .    
473	. T T T T T . T . T . . . . . . . . . . . . . . . . . . . . . . .    
474	. G G G G G . G . G . . . . . . . . . . . . . . . . . . . . . . .    
475	. G G G G G . G . G . . . . . . . . . . . . . . . . . . . . . . .    
476	. T T T T T . T . T . . . . . . . . . . . . . . . . . . . . . . .    
477	. G G G G G . G . G . . . . . . . . . . . . . . . . . . . . . . .    
478	. G G G G G . G . G . . . . . . . . . . . . . . . . . . . . . . .    
479	. G G G G G . G . G . . . . . . . . . . . . . . . . . . . . . . .    
480	. C C C C C . C . C . . . . . . . . . . . . . . . . . . . . . . .    
481	. A A A A A . A . A . . . . . . . . . . . . . . . . . . . . . . .    
482	. A A A A A . A . A . . . . . . . . . . . . . . . . . . . . . . .    
483	. G C C C C . C . C . . . . . . . . . . . . . . . . . . . . . . .    
484	. . A A A A . A . A . . . . . . . . . . . . . . . . . . . . . . .    
485	. . A A A A . A . A . . . . . . . . . . . . . . . . . . . . . . .    
486	. . G G G G . G . G . . . . . . . . . . . . . . . . . . . . . . .    
487	. . T T T T . T . T . . . . . . . . . . . . . . . . . . . . . . .    
488	. . G G G G . G . G . . . . . . . . . . . . . . . . . . . . . . .    
489	. . T T T T . T . T . . . . . . . . . . . . . . . . . . . . . . .    
490	. . G G G G . G . G . . . . . . . . . . . . . . . . . . . . . . .    
491	. . A A A A . A . A . . . . . . . . . . . . . . . . . . . . . . .    
492	. . C C C C . C . C . . . . . . . . . . . . . . . . . . . . . . .    
493	. . C C C C . C . C . . . . . . . . . . . . . . . . . . . . . . .    
494	. . T T T T . T . T . . . . . . . . . . . . . . . . . . . . . . .    
495	. . G G G G . G . G . . . . . . . . . . . . . . . . . . . . . . .    
496	. . G G G G . G . G . . . . . . . . . . . . . . . . . . . . . . .    
497	. . A A A A . A . A . . . . . . . . . . . . . . . . . . . . . . .    
498	. . G G G G . G . G . . . . . . . . . . . . . . . . . . . . . . .    
499	. . G G G G . G . G . . . . . . . . . . . . . . . . . . . . . . .    
500	. . A A A A . A . A . . . . . . . . . . . . . . . . . . . . . . .    
501	. . T T T T . T . T . . . . . . . . . . . . . . . . . . . . . . .    
502	. . G G G G . G . G . . . . . . . . . . . . . . . . . . . . . . .    
503	. . A A A A . A . A . . . . . . . . . . . . . . . . . . . . . . .    
504	. . G G G G . G . G . . . . . . . . . . . . . . . . . . . . . . .    
505	. . C C C C . C . C . . . . . . . . . . . . . . . . . . . . . . .    
506	. . G G G G . G . G . . . . . . . . . . . . . . . . . . . . . . .    
507	. . T T T T . T . T . . . . . . . . . . . . . . . . . . . . . . .    
508	. . G G G G . G . G . . . . . . . . . . . . . . . . . . . . . . .    
509	. . T T T T . T . T . . . . . . . . . . . . . . . . . . . . . . .    
510	. . G G G G . G . G . . . . . . . . . . . . . . . . . . . . . . .    
511	. . G G G G . G . G . . . . . . . . . . . . . . . . . . . . . . .    
512	. . T T T T . T . T . . . . . . . . . . . . . . . . . . . . . . .    
513	. . G G G G . G . G . . . . . . . . . . . . . . . . . . . . . . .    
514	. . G G G G . G . G . . . . . . . . . . . . . . . . . . . . . . .    
515	. . G G G G . G . G . . . . . . . . . . . . . . . . . . . . . . .    
516	. . C C C C . C . C . . . . . . . . . . . . . . . . . . . . . . .    
517	. . A A A A . A . A . . . . . . . . . . . . . . . . . . . . . . .    
518	. . A A A A . A . A . . . . . . . . . . . . . . . . . . . . . . .    
519	. . G G G G . G . G . . . . . . . . . . . . . . . . . . . . . . .    
520	. . G G G G . G . G . . . . . . . . . . . . . . . . . . . . . . .    
521	. . A A A A . A . A . . . . . . . . . . . . . . . . . . . . . . .    
522	. . G G G G . G . G . . . . . . . . . . . . . . . . . . . . . . .    
523	. . C C C C . C . C . . . . . . . . . . . . . . . . . . . . . . .    
524	. . A A A A . A . A . . . . . . . . . . . . . . . . . . . . . . .    
525	. . G G G G . G . G . . . . . . . . . . . . . . . . . . . . . . .    
526	. . G G G G . G . G . . . . . . . . . . . . . . . . . . . . . . .    
527	. . G G G G . G . G . . . . . . . . . . . . . . . . . . . . . . .    
528	. . T T T T . T . T . . . . . . . . . . . . . . . . . . . . . . .    
529	. . C C C C . C . C . . . . . . . . . . . . . . . . . . . . . . .    
530	. . A A A A . G . G . . . . . . . . . . . . . . . . . . . . . . .    
531	. . G G G G . G . G . . . . . . . . . . . . . . . . . . . . . . .    
532	. . A A A A . A . A . . . . . . . . . . . . . . . . . . . . . . .    
533	. . A A A A . A . A . . . . . . . . . . . . . . . . . . . . . . .    
534	. . C C C C . C . C . . . . . . . . . . . . . . . . . . . . . . .    
535	. . C C C C . C . C . . . . . . . . . . . . . . . . . . . . . . .    
536	. . T T T T . T . T . . . . . . . . . . . . . . . . . . . . . . .    
537	. . G G G G . G . G . . . . . . . . . . . . . . . . . . . . . . .    
538	. . G G G G . G . G . . . . . . . . . . . . . . . . . . . . . . .    
539	. . C C C C . C . C . . . . . . . . . . . . . . . . . . . . . . .    
540	. . C C C C . C . C . . . . . . . . . . . . . . . . . . . . . . .    
541	. . A A A A . A . A . . . . . . . . . . . . . . . . . . . . . . .    
542	. . G G G G . G . G . . . . . . . . . . . . . . . . . . . . . . .    
543	. . A A A A . A . A . . . . . . . . . . . . . . . . . . . . . . .    
544	. . C C C C . C . C . . . . . . . . . . . . . . . . . . . . . . .    
545	. . A A A A . A . A . . . . . . . . . . . . . . . . . . . . . . .    
546	. . G G G G . G . G . . . . . . . . . . . . . . . . . . . . . . .    
547	. . T T T T . T . T . . . . . . . . . . . . . . . . . . . . . . .    
548	. . G G G G . G . G . . . . . . . . . . . . . . . . . . . . . . .    
549	. . G G G G . G . G . . . . . . . . . . . . . . . . . . . . . . .    
550	. . A A A A . A . A . . . . . . . . . . . . . . . . . . . . . . .    
551	. . A A A A . A . A . . . . . . . . . . . . . . . . . . . . . . .    
552	. . C C C C . C . C . . . . . . . . . . . . . . . . . . . . . . .    
553	. . A A A A . A . A . . . . . . . . . . . . . . . . . . . . . . .    
554	. . A A A A . A . A . . . . . . . . . . . . . . . . . . . . . . .    
555	. . C C C C . C . C . . . . . . . . . . . . . . . . . . . . . . .    
556	. . T T T T T T . T . . . . . . . . . . . . . . . . . . . . . . .    
557	. . G G G G G G . G . . . . . . . . . . . . . . . . . . . . . . .    
558	. . T T T T T T . T . . . . . . . . . . . . . . . . . . . . . . .    
559	. . G G G G G G . G . . . . . . . . . . . . . . . . . . . . . . .    
560	. . C C C C C C . C . . . . . . . . . . . . . . . . . . . . . . .    
561	. . C C C C C C . C . . . . . . . . . . . . . . . . . . . . . . .    
562	. . T T T T T T . T . . . . . . . . . . . . . . . . . . . . . . .    
563	. . T T T T T T . T . . . . . . . . . . . . . . . . . . . . . . .    
564	. . T T T T T T . T . . . . . . . . . . . . . . . . . . . . . . .    
565	. . C C C C C C . C . . . . . . . . . . . . . . . . . . . . . . .    
566	. . T T T T T T . T . . . . . . . . . . . . . . . . . . . . . . .    
567	. . A A A A A A . A . . . . . . . . . . . . . . . . . . . . . . .    
568	. . G G G G G G . G . . . . . . . . . . . . . . . . . . . . . . .    
569	. . A A A A A A . A . . . . . . . . . . . . . . . . . . . . . . .    
570	. . G G G G G G . G . . . . . . . . . . . . . . . . . . . . . . .    
571	. . T T T T T T . T . . . . . . . . . . . . . . . . . . . . . . .    
572	. . C C C C C C . C . . . . . . . . . . . . . . . . . . . . . . .    
573	. . C C C C C C . C . . . . . . . . . . . . . . . . . . . . . . .    
574	. . T T T T T T . T . . . . . . . . . . . . . . . . . . . . . . .    
575	. . C C C C C C . C . . . . . . . . . . . . . . . . . . . . . . .    
576	. . A A A A A A . A . . . . . . . . . . . . . . . . . . . . . . .    
577	. . G G G G G G . G . . . . . . . . . . . . . . . . . . . . . . .    
578	. . C C C C C C . C . . . . . . . . . . . . . . . . . . . . . . .    
579	. . T T T T T T . T . . . . . . . . . . . . . . . . . . . . . . .    
580	. . A A A A A A . A . . . . . . . . . . . . . . . . . . . . . . .    
581	. . A A A A A A . A . . . . . . . . . . . . . . . . . . . . . . .    
582	. . A A A A A A . A . . . . . . . . . . . . . . . . . . . . . . .    
583	. . T T T T T T . T . . . . . . . . . . . . . . . . . . . . . . .    
584	. . C C C C C C . C . . . . . . . . . . . . . . . . . . . . . . .    
585	. . A A A A A A . A . . . . . . . . . . . . . . . . . . . . . . .    
586	. . A A A A A A . A . . . . . . . . . . . . . . . . . . . . . . .    
587	. . A A A A A A . A . . . . . . . . . . . . . . . . . . . . . . .    
588	. . G G G G G G . G . . . . . . . . . . . . . . . . . . . . . . .    
589	. . A A A A A A . A . . . . . . . . . . . . . . . . . . . . . . .    
590	. . T T T T T T . T . . . . . . . . . . . . . . . . . . . . . . .    
591	. . C C C C C C . C . . . . . . . . . . . . . . . . . . . . . . .    
592	. . A A A A A A . A . . . . . . . . . . . . . . . . . . . . . . .    
593	. . A A A A A A . A . . . . . . . . . . . . . . . . . . . . . . .    
594	. . C C C C C C . C . . . . . . . . . . . . . . . . . . . . . . .    
595	. . G G G G G G . G . . . . . . . . . . . . . . . . . . . . . . .    
596	. . T T T T T T . T . . . . . . . . . . . . . . . . . . . . . . .    
597	. . T T T T T T . T . . . . . . . . . . . . . . . . . . . . . . .    
598	. . A A A A A A . A . . . . . . . . . . . . . . . . . . . . . . .    
599	. . A A A A A A . A . . . . . . . . . . . . . . . . . . . . . . .    
600	. . T T T T T T . T . . . . . . . . . . . . . . . . . . . . . . .    
601	. . G G G G G G . G . . . . . . . . . . . . . . . . . . . . . . .    
602	. . A A A A A A . A . . . . . . . . . . . . . . . . . . . . . . .    
603	. . G G G G G G . G . . . . . . . . . . . . . . . . . . . . . . .    
604	. . A A A A A A . A . . . . . . . . . . . . . . . . . . . . . . .    
605	. . T T T T T T . T . . . . . . . . . . . . . . . . . . . . . . .    
606	. . T T T T T T . T . . . . . . . . . . . . . . . . . . . . . . .    
607	. . T T T T T T . T . . . . . . . . . . . . . . . . . . . . . . .    
608	. . T T T T T T . T . . . . . . . . . . . . . . . . . . . . . . .    
609	. . C C C C C C . C . . . . . . . . . . . . . . . . . . . . . . .    
610	. . T T T T T T . T . . . . . T . . . . . . . . . . . . . . . . .    
611	. . A A A A A A . A . . . . . A . . . . . . . . . . . . . . . . .    
612	. . T T T T T T . T . . . . . T . . . . . . . . . . . . . . . . .    
613	. . G G G G G G . G . . . . . G . . . . . . . . . . . . . . . . .    
614	. . A A A A A A . A . . . . . A . . . . . . . . . . . . . . . . .    
615	. . C C C C C C . C . . . . . C . . . . . . . . . . . . . . . . .    
616	. . C C C C C C . C . . . . . C . . . . . . . . . . . . . . . . .    
617	. . T T T T T T . T . . . . . T . . . . . . . . . . . . . . . . .    
618	. . G G G G G G . G . . . . . G . . . . . . . . . . . . . . . . .    
619	. . G G G G G G . G . . . . . G . . . . . . . . . . . . . . . . .    
620	. . T T T T T T . T . . . . . T . . . . . . . . . . . . . . . . .    
621	. . C C C C C C . C . . . . . C . . . . . . . . . . . . . . . . .    
622	. . A A A A A A . A . . . . . A . . . . . . . . . . . . . . . . .    
623	. . G G G G G G . G . . . . . G . . . . . . . . . . . . . . . . .    
624	. . A A A A A A . A . . . . . A . . . . . . . . . . . . . . . . .    
625	. . C C C C C C . C . . . . . C . . . . . . . . . . . . . . . . .    
626	. . A A A A A A . A . . . . . A . . . . . . . . . . . . . . . . .    
627	. . G G G G G G . G . . . . . G . . . . . . . . . . . . . . . . .    
628	. . A A A A A A . A . . . . . A . . . . . . . . . . . . . . . . .    
629	. . T T T T T T . T . . . . . T . . . . . . . . . . . . . . . . .    
630	. . C C C C C C . C . . . . . C . . . . . . . . . . . . . . . . .    
631	. . A A A A A A . A . . . . . A . . . . . . . . . . . . . . . . .    
632	. . A A A A A A . A . . . . . A . . . . . . . . . . . . . . . . .    
633	. . T T T T T T . T . . . . . T . . . . . . . . . . . . . . . . .    
634	. . A A A A A A . A . . . . . A . . . . . . . . . . . . . . . . .    
635	. . G G G G G G . G . . . . . G . . . . . . . . . . . . . . . . .    
636	. . G G G G G G . G . . . . . G . . . . . . . . . . . . . . . . .    
637	. . A A A A A A . A . . . . . A . . . . . . . . . . . . . . . . .    
638	. . A A A A A A . A . . . . . A . . . . . . . . . . . . . . . . .    
639	. . A A A A A A . A . A . . . A . . . . . . . . . . . . . . . . .    
640	. . A A A A A A . A . A . . . A . . . . . . . . . . . . . . . . .    
641	. . C C C C C C . C . C . . . C . . . . . . . . . . . . . . . . .    
642	. . A A A A A A . A . A . . . A . . . . . . . . . . . . . . . . .    
643	. . C C C C C C . C . C . . . C . . . . . . . . . . . . . . . . .    
644	. . C C C C C C . C . C . . . C . . . . . . . . . . . . . . . . .    
645	. . G G G G G G . G . G . . . G G . . . . . . . . . . . . . . . .    
646	. . A A A A A A . A . A . . . A A . . . . . . . . . . . . . . . .    
647	. . T T T T T T . T . T . . . T T . . . . . . . . . . . . . . . .    
648	. . A A A A A A . A . A . . . A A . . . . . . . . . . . . . . . .    
649	. . G G G G G G . G . G . . . G G . . . . . . . . . . . . . . . .    
650	. . A A A A A A A A . A . . . A A . . . . . . . . . . . . . . . .    
651	. . A A A A A A A A . A . . . A A . . . . . . . . . . . . . . . .    
652	. . A A A A A A A A . A . . . A A . . . . . . . . . . . . . . . .    
653	. . A A A A A A A A . A . . . A A . . . . . . . . . . . . . . . .    
654	. . G G G G G G G G . G . . . G G G . . . . . . . . . . . . . . .    
655	. . A A A A A A A A . A . . . A A A . . . . . . . . . . . . . . .    
656	. . A A A A A A A A . A . . . A A A . . . . . . . . . . . . . . .    
657	. . G G G G G G G G . G . . . G G G . . . . . . . . . . . . . . .    
658	. . A A A A A A A A . A . . . A A A . . . . . . . . . . . . . . .    
659	. . A A A A A A A A . A . . . A A A . . . . . . . . . . . . . . .    
660	. . G G G G G G G G . G . . . G G G . . . . . . . . . . . . . . .    
661	. . G G G G G G G G . G . . . G G G . . . . . . . . . . . . . . .    
662	. . A A A A A A A A . A . . . A A A . . . . . . . . . . . . . . .    
663	. . A A A A A A A A . A . . . A A A . . . . . . . . . . . . . . .    
664	. . A A A A A A A A . A . . . A A A . . . . . . . . . . . . . . .    
665	. . A A A A A A A A . A . . . A A A . . . . . . . . . . . . . . .    
666	. . G G G G G G G G . G . . . G G G . . . . . . . . . . . . . . .    
667	. . A A A A A A A A . A . . . A A A . . . . . . . . . . . . . . .    
668	. . A A A A A A A A . A . . . A A A . . . . . . . . . . . . . . .    
669	. . G G G G G G G G . G . . . G G G . . . . . . . . . . . . . . .    
670	. . T T T T T T T T . T . . . T T T . . . . . . . . . . . . . . .    
671	. . C C C C C C C C . C . . . C C C . . . . . . . . . . . . . . .    
672	. . A A A A A A A A . A . . . A A A . . . . . . . . . . . . . . .    
673	. . A A A A A A A A . A . . . A A A . . . . . . . . . . . . . . .    
674	. . A A A A A A A A . A . . . A A A . . . . . . . . . . . . . . .    
675	. . T T T T T T T T . T . . . T T T . . . . . . . . . . . . . . .    
676	. . T T T T T T T T . T . . . T T T . . . . . . . . . . . . . . .    
677	. . G G G G G G G G . G . . . G G G . . . . . . . . . . . . . . .    
678	. . C C C C C C C C . C . . . C C C . . . . . . . . . . . . . . .    
679	. . A A A A A A A A . A . . . A A A . . . . . . . . . . . . . . .    
680	. . C C C C C C C C . C . . . C C C . . . . . . . . . . . . . . .    
681	. . A A A A A A A A . A . . . A A A . . . . . . . . . . . . . . .    
682	. . C C C C C C C C . C . . . C C C . . . . . . . . . . . . . . .    
683	. . T T T T T T T T . T . . . T T T . . . . . . . . . . . . . . .    
684	. . G G G G G G G G . G . . . G G G . . . . . . . . . . . . . . .    
685	. . C C C C C C C C . C . . . C C C . . . . . . . . . . . . . . .    
686	. . T T T T T T T T . T . . . T T T . . . . . . . . . . . . . . .    
687	. . C C C C C C C C . C . . . C C C . . . . . . . . . . . . . . .    
688	. . T T T T T T T T . T . . . T T T . . . . . . . . . . . . . . .    
689	. . G G G G G G G G . G . . . G G G . . . . . . . . . . . . . . .    
690	. . A A A A A A A A . A . . . A A A . . . . . . . . . . . . . . .    
691	. . A A A A A A A A . A . . . A A A . . . . . . . . . . . . . . .    
692	. . G G G G G G G G . G . . . G G G . . . . . . . . . . . . . . .    
693	. . C C C C C C C C . C . . . C C C . . . . . . . . . . . . . . .    
694	. . T T T T T T T T . T . . . T T T . . . . . . . . . . . . . . .    
695	. . C C C C C C C C . C . . . C C C . . . . . . . . . . . . . . .    
696	. . C C C C C C C C . C . . . C C C . . . . . . . . . . . . . . .    
697	. . C C C C C C C C . C . . . C C C . . . . . . . . . . . . . . .    
698	. . C C C C C C C C . C . . . C C C . . . . . . . . . . . . . . .    
699	. . C C C C C C C C . C . . . C C C . . . . . . . . . . . . . . .    
700	. . T T T T T T T T . T . . . T T T . . . . . . . . . . . . . . .    
701	. . T T T T T T T T . T . . . T T T . . . . . . . . . . . . . . .    
702	. . A A A A A A A A . A . . . A A A . . . . . . . . . . . . . . .    
703	. . C C C C C C C C . C . . . C C C . . . . . . . . . . . . . . .    
704	. . A A A A A A A A . A . . . A A A . . . . . . . . . . . . . . .    
705	. . G G G G G G G G . G . . . G G G . . . . . . . . . . . . . . .    
706	. . C C C C C C C C . C . . . C C C . . . . . . . . . . . . . . .    
707	. . A A A A A A A A . A . . . A A A . . . . . . . . . . . . . . .    
708	. . G G G G G G G G . G . . . G G G . . . . . . . . . . . . . . .    
709	. . C C C C C C C C . C . . . C C C . . . . . . . . . . . . . . .    
710	. . T T T T T T T T . T . . . T T T . . . . . . . . . . . . . . .    
711	. . C C C C C C C C . C . . . C C C . . . . . . . . . . . . . . .    
712	. . T T T T T T T T . T T . . T T T . . . . . . . . . . . . . . .    
713	. . G G G G G G G G . G G G . G G G . . . . G . . . . . . . . . .    
714	. . A A A A A A A A . A A A . A A A . . . . A . . . . . . . . . .    
715	. . G G G G G G G G . G G G . G G G . . . . G . . . . . . . . . .    
716	. . C C C C C C C C . C C C . C C C . . . . C . . . . . . . . . .    
717	. . C C C C C C C C . C C C . C C C C . . . C C . . . . . . . . .    
718	. . A A A A A A A A . A A A . A A A A . . . A A . . . . . . . . .    
719	. . G G G G G G G G . G G G . G G G G . . . G G . . . . . . . . .    
720	. . G G G G G G G G . G G G . G G G G . . . G G . . . . . . . . .    
721	. . A A A A A A A A . A A A . A A A A . . . A A . . . . . . . . .    
722	. . A A A A A A A A . A A A . A A A A . . . A A . . . . . . . . .    
723	. . G G G G G G G G . G G G . G G G G . . . G G . . . . . . . . .    
724	. . C C C C C C C C . C C C . C C C C . . . C C . . . . . . . . .    
725	. . T T T T T T T T . T T T . T T T T . . . T T . . . . . . . . .    
726	. . G G G G G G G G . G G G . G G G G . . . G G . . . . . . . . .    
727	. . C C C C C C C C . C C C . C C C C . C . C C . . . . . . . . .    
728	. . A A A A A A A A . A A A . A A A A . A . A A . . . . . . . . .    
729	. . A A A A A A A A . A A A . A A A A . A . A A . . . . . . . . .    
730	. . T T T T T T T T . T T T . T T T T . T . T T . . . . . . . . .    
731	. . A A A A A A A A . A A A . A A A A . A . A A . . . . . . . . .    
732	. . C C C C C C C C . C C C . C C C C . C . C C . . . . . . . . .    
733	. . A A A A A A A A . A A A . A A A A . A . A A . . . . . . . . .    
734	. . A A A A A A A A . A A A . A A A A . A . A A . . . . . . . . .    
735	. . T T T T T T T T . T T T . T T T T . T . T T . . . . . . . . .    
736	. . A A A A A A A A . A A A . A A A A . A . A A . . . . . . . . .    
737	. . C C C C C C C C . C C C . C C C C . C . C C . . . . . . . . .    
738	. . T T T T T T T T . T T T . T T T T . T . T T . . . . . . . . .    
739	. . G G G G G G G G . G G G . G G G G . G . G G . . . . . . . . .    
740	. . G . G G G G G G . G G G . G G G G . G . G G G . . . . . . . .    
741	. . T . T T T T T T . T T T . T T T T . T . T T T . . . . . . . .    
742	. . G . G G G G G G . G G G . G G G G . G . G G G . . . . . . . .    
743	. . C . C C C C C C . C C C . C C C C . C . C C C . . . . . . . .    
744	. . C . C C C C C C . C C C . C C C C . C . C C C . . . . . . . .    
745	. . C . C C C C C C . C C C . C C C C . C . C C C . . . . . . . .    
746	. . A . A A A A A A . A A A . A A A A . A . A A A . . . . . . . .    
747	. . G . G G G G G G . G G G . G G G G . G . G G G . . . . . . . .    
748	. . T . T T T T T T . T T T . T T T T . T . T T T . . . . . . . .    
749	. . T . T T T T T T . T T T . T T T T . T . T T T . . . . . . . .    
750	. . G . G G G G G G . G G G . G G G G . G . G G G . . . . . . . .    
751	. . G . G G G G G G . G G G . G G G G . G . G G G . . . . G . . .    
752	. . A . A A A A A A . A A A . A A A A . A . A A A . . . . A . . .    
753	. . A . A A A A A A . A A A . A A A A . A . A A A . . . . A . . .    
754	. . C . C C C C C C . C C C . C C C C . C . C C C . . . . C . . .    
755	. . G . G G G G G G . G G G . G G G G . G . G G G . . . G G . . .    
756	. . T . T T T T T T . T T T . T T T T . T . T T T . . . T T . . .    
757	. . T . T T T T T T . T T T . T T T T . T . T T T . . . T T . . .    
758	. . T . T T T T T T . T T T . T T T T . T . T T T . . . T T . . .    
759	. . C . C C C C C C . C C C . C C C C . C . C C C . . . C C . . .    
760	. . A . A A A A A A . A A A . A A A A . A . A A A . . . A A . . .    
761	. . G . G G G G G G . G G G . G G G G . G . G G G . . . G G . . .    
762	. . C . C C C C C C . C C C . C C C C . C . C C C . . . C C . . .    
763	. . A . A A A A A A . A A A . A A A A . A . A A A . . . A A . . .    
764	. . T . T T T T T T . T T T . T T T T . T . T T T . . . T T . . .    
765	. . T . T T T T T T . T T T . T T T T . T . T T T . . . T T . . .    
766	. . T . T T T T T T . T T T . T T T T . T . T T T . . . T T . . .    
767	. . C . C C C C C C . C C C . C C C C . C . C C C . . . C C . . .    
768	. . A . A A A A A A . A A A . A A A A . A . A A A . . . A A . . .    
769	. . A . A A A A A A . A A A . A A A A . A . A A A . A . A A . . .    
770	. . A . G G G G G G . G G G . G G G G . G . G G G . G . G G . . .    
771	. . . . C C C C C C . C C C . C C C C . C . C C C . C . C C . . .    
772	. . . . C C C C C C . C C C . C C C C . C . C C C . C . C C . . .    
773	. . . . C C C C C C . C C C . C C C C . C . C C C . C . C C . . .    
774	. . . . T T . T T T . T T T . T T T T . T . T T T . T . T T . . .    
775	. . . . T T . T T T . T T T . T T T T . T . T T T . T . T T . . .    
776	. . . . T . . T T T . T T T . T T T T . T . T T T . T . T T . . .    
777	. . . . T . . C C C . C C C . C C C C . C . C C C . C . C C C . .    
778	. . . . . . . A A A . A A A . A A A A . A . A A A . A . A A A . .    
779	. . . . . . . A A A . A A A A A A A A . A . A A A . A . A A A . .    
780	. . . . . . . C C C . C C C C C C C C . C . C C C . C . C C C . .    
781	. . . . . . . A A A . A A A A A A A A . A . A A A . A . A A A . .    
782	. . . . . . . A A A . A A A A A A A A . A . A A A . A . A A A . .    
783	. . . . . . . C C C . C C C C C C C C . C . C C C . C . C C C . .    
784	. . . . . . . G G G . G G G G G G G G . G . G G G . G . G G G . .    
785	. . . . . . . A A A . A A A A A A A A . A . A A A . A . A A A . .    
786	. . . . . . . A A A . A A A A A A A A . A . A A A . A . A A A . .    
787	. . . . . . . T T T . T T T T T T T T . T . T T T . T . T T T . .    
788	. . . . . . . G G G . G G G G G G G G . G . G G G . G . G G G . .    
789	. . . . . . . G G G . G G G G G G G G . G . G G G . G . G G G . .    
790	. . . . . . . A A A . A A A A A A A A . A . A A A . A . A A A . .    
791	. . . . . . . G G G . G G G G G G G G . G . G G G . G . G G G . .    
792	. . . . . . . A A A . A A A A A A A A . A . A A A . A . A A A . .    
793	. . . . . . . T T T . T T T T T T T T . T . T T T . T . T T T . .    
794	. . . . . . . G G G . G G G G G G G G . G . G G G . G . G G G . .    
795	. . . . . . . T T T . T T T T T T T T . T . T T T . T . T T T . .    
796	. . . . . . . T N T . T T T T T T T T . T . T T T . T . T T T . .    
797	. . . . . . . T T T . T T T T T T T T . T . T T T . T . T T T . .    
798	. . . . . . . G G G . G G G G G G G G . G . G G G . G . G G G . .    
799	. . . . . . . A A A . A A A A A A A A . A . A A A . A . A A A . .    
800	. . . . . . . C C C . C C C C C C C C . C . C C C . C . C C C . .    
801	. . . . . . . A A A . A A A A A A A A . A . A A A . A . A A A . .    
802	. . . . . . . A A A . A A A A A A A A . A . A A A . A . A A A . .    
803	. . . . . . . T T T . T T T T T T T T . T . T T T . T . T T T . .    
804	. . . . . . . A A A . A A A A A A A A . A . A A A . A . A A A . .    
805	. . . . . . . G G G . G G G G G G G G . G . G G G . G . G G G . .    
806	. . . . . . . C C C . C C C C C C C C . C . C C C . C . C C C . .    
807	. . . . . . . A A A . A A A A A A A A . A . A A A . A . A A A . .    
808	. . . . . . . G G G . G G G G G G G G . G . G G G . G . G G G . .    
809	. . . . . . . A A A . A A A A A A A A . A . A A A . A . A A A . .    
810	. . . . . . . A A A . A A A A A A A A . A . A A A . A . A A A . .    
811	. . . . . . . A A A . A A A A A A A A . A . A A A . A . A A A . .    
812	. . . . . . . A A A . A A A A A A A A . A . A A A . A . A A A . .    
813	. . . . . . . G G G . G G G G G G G G . G . G G G . G . G G G . .    
814	. . . . . . . T T T . T T T T T T T T . T . T T T . T . T T T . .    
815	. . . . . . . A A A . A A A A A A A A . A . A A A . A . A A A . .    
816	. . . . . . . A A A . A A A A A A A A . A . A A A . A . A A A . .    
817	. . . . . . . T T T . T T T T T T T T . T . T T T . T . T T T . .    
818	. . . . . . . T T T . T T T T T T T T . T . T T T . T . T T T . .    
819	. . . . . . . C C C . C C C C C C C C . C . C C C . C . C C C . .    
820	. . . . . . . T T T . T T T T T T T T . T . T T T . T . T T T . .    
821	. . . . . . . C C C . C C C C C C C C . C . C C C . C . C C C . .    
822	. . . . . . . T T T . T T T T T T T T . T . T T T . T . T T T . .    
823	. . . . . . . A A A . A A A A A A A A . A . A A A . A . A A A . .    
824	. . . . . . . C C C . C C C C C C C C . C . C C C . C . C C C . .    
825	. . . . . . . T T T . T T T T T T T T . T . T T T . T . T T T . .    
826	. . . . . . . T T T . T T T T T T T T . T . T T T . T . T T T . .    
827	. . . . . . . C C C . C C C C C C C C . C . C C C . C . C C C . .    
828	. . . . . . . A A A . A A A A A A A A . A . A A A . A . A A A . .    
829	. . . . . . . C C C . C C C C C C C C . C . C C C . C C C C C . .    
830	. . . . . . . C C C . C C C C C C C C . C . C C C . C C C C C . .    
831	. . . . . . . A A A . A A A A A A A A . A . A A A . A A A A A . .    
832	. . . . . . . C C C . C C C C C C C C . C . C C C . C C C C C . .    
833	. . . . . . . T T T . T T T T T T T T . T . T T T . T T T T T . .    
834	. . . . . . . C C C . C C C C C C C C . C . C C C . C C C C C . .    
835	. . . . . . . T T T . T T T T T T T T . T . T T T . T T T T T . .    
836	. . . . . . . A A A . A A A A A A A A . A . A A A . A A A A A . .    
837	. . . . . . . A A A . A A A A A A A A . A . A A A . A A A A A . .    
838	. . . . . . . T T T . T T T T T T T T . T T T T T . T T T T T . .    
839	. . . . . . . A A A . A A A A A A A A . A A A A A . A A A A A . .    
840	. . . . . . . A A A . A A A A A A A A . A A A A A . A A A A A . .    
841	. . . . . . . T T T . T T T T T T T T . T T T T T . T T T T T . .    
842	. . . . . . . G G G . G G G G G G G G . G G G G G . G G G G G . .    
843	. . . . . . . G G G . G G G G G G G G . G G G G G . G G G G G . .    
844	. . . . . . . A A A . A A A A A A A A . A A A A A . A A A A A . .    
845	. . . . . . . G G G . G G G G G G G G . G G G G G . G G G G G . .    
846	. . . . . . . G G G . G G G G G G G G . G G G G G . G G G G G . .    
847	. . . . . . . A A A . A A A A A A A A . A A A A A . A A A A A . .    
848	. . . . . . . T T T . T T T T T T T T . T T T T T . T T T T T . .    
849	. . . . . . . T T T . T T T T T T T T . T T T T T . T T T T T . .    
850	. . . . . . . C C C . C C C C C C C C . C C C C C . C C C C C . .    
851	. . . . . . . T T T . T T T T T T T T . T T T T T . T T T T T . .    
852	. . . . . . . T T T . T T T T T T T T . T T T T T . T T T T T . .    
853	. . . . . . . C C C . C C C C C C C C . C C C C C . C C C C C . .    
854	. . . . . . . A A A . A A A A A A A A . A A A A A . A A A A A . .    
855	. . . . . . . T T T . T T T T T T T T . T T T T T . T T T T T . .    
856	. . . . . . . G G G . G G G G G G G G . G G G G G . G G G G G . .    
857	. . . . . . . G G G . G G G G G G G G . G G G G G . G G G G G . .    
858	. . . . . . . A A A . A A A A A A A A . A A A A A . A A A A A . .    
859	. . . . . . . T T T . T T T T T T T T . T T T T T . T T T T T . .    
860	. . . . . . . A A A . A A A A A A A A . A A A A A . A A A A A . .    
861	. . . . . . . C C C . C C C C C C C C . C C C C C . C C C C C . .    
862	. . . . . . . T T T . T T T T T T T T . T T T T T . T T T T T . .    
863	. . . . . . . C C C . C C C C C C C C . C C C C C . C C C C C . .    
864	. . . . . . . T T T . T T T T T T T T . T T T T T . T T T T T . .    
865	. . . . . . . T T T . T T T T T T T T . T T T T T . T T T T T . .    
866	. . . . . . . T T T . T T T T T T T T . T T T T T . T T T T T . .    
867	. . . . . . . G G G . G G G G G G G G . G G G G G . G G G G G . .    
868	. . . . . . . C C C . C C C C C C C C . C C C C C . C C C C C . .    
869	. . . . . . . T T T . T T T T T T T T . T T T T T . T T T T T . .    
870	. . . . . . . T T T . T T T T T T T T . T T T T T . T T T T T . .    
871	. . . . . . . C C C . C C C C C C C C . C C C C C . C C C C C . .    
872	. . . . . . . G G G . G G G G G G G C . G G G G G . G G G G G . .    
873	. . . . . . . T T T . T T T T T T T T . T T T T T . T T T T T . .    
874	. . . . . . . A A A . A A A A A A A A . A A A A A . A A A A A . .    
875	. . . . . . . A A A . A A A A A A A A . A A A A A . A A A A A . .    
876	. . . . . . . T T T . T T T T T T T T . T T T T T . T T T T T . .    
877	. . . . . . . G G G . G G G G G G G G . G G G G G . G G G G G . .    
878	. . . . . . . G G G . G G G G G G G G . G G G G G . G G G G G . .    
879	. . . . . . . T T T . T T T T T T T T . T T T T T . T T T T T . .    
880	. . . . . . . T T T . T T T T T T T T . T T T T T . T T T T T . .    
881	. . . . . . . A A A . A A A A A A A A . A A A A A . A A A A A . .    
882	. . . . . . . A A A . A A A A A A A A . A A A A A . A A A A A . .    
883	. . . . . . . G G G . G G G G G G G G . G G G G G . G G G G G . .    
884	. . . . . . . A A A . A A A A A A A A . A A A A A . A A A A A . .    
885	. . . . . . . A A A . A A A A A A A A . A A A A A . A A A A A . .    
886	. . . . . . . A A A . A A A A A A A A . A A A A A . A A A A A . .    
887	. . . . . . . T T T . T T T T T T T T . T T T T T . T T T T T . .    
888	. . . . . . . T G T G T G G G G G T T . G G G T G . G T G G G . .  7	 transversion  
889	. . . . . . . C C C C C C C C C C C C . C C C C C . C C C C C . .    
890	. . . . . . . G A G A A A A A A A A A . A A A A A . A G A A A . .    
891	. . . . . . . G G G G G G G G G G G G . G G G G G . G G G G G . .    
892	. . . . . . . A A A A A A A A A A A A . A A A A A . A A A A A . .    
893	. . . . . . . C C C C C C C C C C C C . C C C C C . C C C C C . .    
894	. . . . . . . C C C C C C C C C C C C . C C C C C . C C C C C . .    
895	. . . . . . . T T T T T T T T T T T T . T T T T T . T T T T T . .    
896	. . . . . . . T T T T T T T T T T T T . T T T T T . T T T T T . .    
897	. . . . . . . T T T T T T T T T T T T . T T T T T . T T T T T . .    
898	. . . . . . . T T T T T T T T T T T T . T T T T T . T T T T T . .    
899	. . . . . . . T T T T T T T T T T T T . T T T T T . T T T T T . .    
900	. . . . . . . C C C C C C C C C C C C . C C C C C . C C C C C . .    
901	. . . . . . . T T T T T T T T T T T T . T T T T T . T T T T T . .    
902	. . . . . . . C C C C C C C C C C C C . C C C C C . C C C C C . .    
903	. . . . . . . T T T T T T T T T T T T . T T T T T . T T T T T . .    
904	. . . . . . . C C C C C C C C C C C C . C C C C C . C C C C C . .    
905	. . . . . . . T T T T T T T T T T T T T T T T T T . T T T T T . .    
906	. . . . . . . T T T T T T T T T T T T T T T T T T . T T T T T . .    
907	. . . . . . . T T T T T T T T T T T T T T T T T T . T T T T T . .    
908	. . . . . . . T T T T T T T T T T T T T T T T T T . T T T T T . .    
909	. . . . . . . T T T T T T T T T T T T T T T T T T . T T T T T . .    
910	. . . . . . . C C C C C C C C C C C C C C C C C C . C C C C C . .    
911	. . . . . . . A A A A A A A A A A A A A A A A A A . A A A A A . .    
912	. . . . . . . T T T T T T T T T T T T T T T T T T . T T T T T . .    
913	. . . . . . . T T T T T T T T T T T T T T T T T T . T T T T T . .    
914	. . . . . . . T T T T T T T T T T T T T T T T T T . T T T T T . .    
915	. . . . . . . T T T T T T T T T T T T T T T T T T . T T T T T . .    
916	. . . . . . . G G G G G G G G G G G G G G G G G G . G G G G G . G    
917	. . . . . . . T T T T T T T T T T T T T T T T T T . T T T T T . T    
918	. . . . . . . C C C C C C C C C C C C C C C C C C . C C C C C . C    
919	. . . . . . . A A A A A A A A A A A A A A A A A A . A A A A A . A    
920	. . . . . . . A A A A A A A A A A A A A A A A A A . A A A A A . A    
921	. . . . . . . T T T T T T T T T T T T T T T T T T . T T T T T . T    
922	. . . . . . . G G G G G G G G G G G G G G G G G G . G G G G G . G    
923	. . . . . . . G G G G G G G G G G G G G G G G G G . G G G G G . G    
924	. . . . . . . G G G G G G G G G G G G G G G G G G . G G G G G . G    
925	. . . . . . . A A A A A A A A A A A A A A A A A A . A A A A A . A    
926	. . . . . . . C C C C C C C C C C C C C C C C C C . C C C C C . C    
927	. . . . . . . A A A A A A A A A A A A A A A A A A . A A A A A . A    
928	. . . . . . . G G G G G G G G G G G G G G G G G G . G G G G G . G    
929	. . . . . . . C C C C C C C C C C C C C C C C C C . C C C C C . C    
930	. . . . . . . C C C C C C C C C C C C C C C C C C . C C C C C . C    
931	. . . . . . . T T T T T T T T T T T T T T T T T T . T T T T T . T    
932	. . . . . . . T T T T T T T T T T T T T T T T T T . T T T T T T T    
933	. . . . . . . G G G G G G G G G G G G G G G G G G . G G G G G G G    
934	. . . . . . . T T T T T T T T T T T T T T T T T T . T T T T T T T    
935	. . . . . . . G G G G G G G G G G G G G G G G G G . G G G G G G G    
936	. . . . . . . A A A A A A A A A A A A A A A A A A . A A A A A A A    
937	. . . . . . . T T T T T T T T T T T T T T T T T T . T T T T T T T    
938	. . . . . . . G G G G G G G G G G G G G G G G G G . G G G G G G G    
939	. . . . . . . A A A A A A A A A A A A A A A A A A . A A A A A A A    
940	. . . . . . . A A A A A A A A A A A A A A A A A A . A A A A A A A    
941	. . . . . . . A A A A A A A A A A A A A A A A A A . A A A A A A A    
942	. . . . . . . A A A A A A A A A A A A A A A A A A . A A A A A A A    
943	. . . . . . . G G G G G G G G G G G G G G G G G G . G G G G G G G    
944	. . . . . . . T T T T T T T T T T T T T T T T T T . T T T T T T T    
945	. . . . . . . T T T T T T T T T T T T T T T T T T . T T T T T T T    
946	. . . . . . . T T T T T T T T T T T T T T T T T T . T T T T T T T    
947	. . . . . . . T T T T T T T T T T T T T T T T T T . T T T T T T T    
948	. . . . . . . C C C C C C C C C C C C C C C C C C . C C C C C C C    
949	. . . . . . . C C C C C C C C C C C C C C C C C C . C C C C C C C    
950	. . . . . . . C C C C C C C C C C C C C C C C C C . C C C C C C C    
951	. . . . . . . T T T T T T T T T T T T T T T T T T . T T T T T T T    
952	. . . . . . . T T T T T T T T T T T T T T T T T T . T T T T T T T    
953	. . . . . . . T T T T T T T T T T T T T T T T T T . T T T T T T T    
954	. . . . . . . C C C C C C C C C C C C C C C C C C . C C C C C C C    
955	. . . . . . . C C C C C C C C C C C C C C C C C C . C C C C C C C    
956	. . . . . . . C C C C C C C C C C C C C C C C C C . C C C C C C C    
957	. . . . . . . C C C C C C C C C C C C C C C C C C . C C C C C C C    
958	. . . . . . . T T T T T T T T T T T T T T T T T T . T T T T T T T    
959	. . . . . . . G G G G G G G G G G G G G G G G G G . G G G G G G G    
960	. . . . . . . T T T T T T T T T T T T T T T T T T . T T T T T T T    
961	. . . . . . . T T T T T T T T T T T T T T T T T T . T T T T T T T    
962	. . . . . . . T T T T T T T T T T T T T T T T T T . T T T T T T T    
963	. . . . . . . T T T T T T T T T T T T T T T T T T . T T T T T T T    
964	. . . . . . . A A A A A A A A A A A A A A A A A A . A A A A A A A    
965	. . . . . . . T T T T T T T T T T T T T T T T T T . T T T T T T T    
966	. . . . . . . A A A A A A A A A A A A A A A A A A . A A A A A A A    
967	. . . . . . . A A A A A A A A A A A A A A A A A A . A A A A A A A    
968	. . . . . . . A A A A A A A A A A A A A A A A A A . A A A A A A A    
969	. . . . . . . T T T T T T T T T T T T T T T T T T . T T T T T T T    
970	. . . . . . . T T T T T T T T T T T T T T T T T T . T T T T T T T    
971	. . . . . . . A A A A A A A A A A A A A A A A A A . A A A A A A A    
972	. . . . . . . C C C C C C C C C C C C C C C C C C . C C C C C C C    
973	. . . . . . . A A A A A A A A A A A A A A A A A A . A A A A A A A    
974	. . . . . . . A A A A A A A A A A A A A A A A A A . A A A A A A A    
975	. . . . . . . T T T T T T T T T T T T T T T T T T . T T T T T T T    
976	. . . . . . . T T T T T T T T T T T T T T T T T T . T T T T T T T    
977	. . . . . . . T T T T T T T T T T T T T T T T T T . T T T T T T T    
978	. . . . . . . A A A A A A A A A A A A A A A A A A . A A A A A A A    
979	. . . . . . . A A A A A A A A A A A A A A A A A A . A A A A A A A    
980	. . . . . . . A A A A A A A A A A A A A A A A A A . A A A A A A A    
981	. . . . . . . A A A A A A A A A A A A A A A A A A . A A A A A A A    
982	. . . . . . . C C C C C C C C C C C C C C C C C C . C C C C C C C    
983	. . . . . . . A A A A A A A A A A A A A A A A A A . A A A A A A A    
984	. . . . . . . A A A A A A A A A A A A A A A A A A . A A A A A A A    
985	. . . . . . . A A A A A A A A A A A A A A A A A A . A A A A A A A    
986	. . . . . . . A A A A A A A A A A A A A A A A A A . A A A A A A A    
987	. . . . . . . A A A A A A A A A A A A A A A A A A . A A A A A A A    
988	. . . . . . . A A A A A A A A A A A A A A A A A A . A A A A A A A    
989	. . . . . . . G G G G G G G G G G G G G G G G G G . G G G G G G G    
990	. . . . . . . A A A A A A A A A A A A A A A A A A . A A A A A A A    
991	. . . . . . . C C C C C C C C C C C C C C C C C C . C C C C C C C    
992	. . . . . . . T T T T T T T T T T T T T T T T T T . T T T T T T T    
993	. . . . . . . A A A A A A A A A A A A A A A A A A . A A A A A A A    
994	. . . . . . . C C C C C C C C C C C C C C C C C C . C C C C C C C    
995	. . . . . . . C C C C C C C C C C C C C C C C C C . C C C C C C C    
996	. . . . . . . A A A A A A A A A A A A A A A A A A . A A A A A A A    
997	. . . . . . . A A A A A A A A A A A A A A A A A A . A A A A A A A    
998	. . . . . . . G G G G G G G G G G G G G G G G G G . G G G G G G G    
999	. . . . . . . C C C C C C C C C C C C C C C C C C . C C C C C C C    
1000	. . . . . . . A A A A A A A A A A A A A A A A A A . A A A A A A A    
1001	. . . . . . . A A A A A A A A A A A A A A A A A A . A A A A A A A    
1002	. . . . . . . T T T T T T T T T T T T T T T T T T . T T T T T T T    
1003	. . . . . . . T T T T T T T T T T T T T T T T T T . T T T T T T T    
1004	. . . . . . . T T T T T T T T T T T T T T T T T T . T T T T T T T    
1005	. . . . . . . A A A A A A A A A A A A A A A A A A . A A A A A A A    
1006	. . . . . . . T T T T T T T T T T T T T T T T T T . T T T T T T T    
1007	. . . . . . . T T T T T T T T T T T T T T T T T T . T T T T T T T    
1008	. . . . . . . G G G G G G G G G G G G G G G G G G . G G G G G G G    
1009	. . . . . . . T T T T T T T T T T T T T T T T T T . T T T T T T T    
1010	. . . . . . . G G G G G G G G G G G G G G G G G G . G G G G G G G    
1011	. . . . . . . G G G G G G G G G G G G G G G G G G . G G G G G G G    
1012	. . . . . . . A A A A A A A A A A A A A A A A A A . A A A A A A A    
1013	. . . . . . . T T T T T T T T T T T T T T T T T T . T T T T T T T    
1014	. . . . . . . T T T T T T T T T T T T T T T T T T . T T T T T T T    
1015	. . . . . . . T T T T T T T T T T T T T T T T T T . T T T T T T T    
1016	. . . . . . . G G G G G G G G G G G G G G G G G G . G G G G G G G    
1017	. . . . . . . G G G G G G G G G G G G G G G G G G . G G G G G G G    
1018	. . . . . . . A A A A A A A A A A A A A A A A A A . A A A A A A A    
1019	. . . . . . . C C C C C C C C C C C C C C C C C C . C C C C C C C    
1020	. . . . . . . A A A A A A A A A A A A A A A A A A . A A A A A A A    
1021	. . . . . . . A A A A A A A A A A A A A A A A A A . A A A A A A A    
1022	. . . . . . . T T T T T T T T T T T T T T T T T T . T T T T T T T    
1023	. . . . . . . T T T T T T T T T T T T T T T T T T . T T T T T T T    
1024	. . . . . . . A A A A A A A A A A A A A A A A A A . A A A A A A A    
1025	. . . . . . . G G G G G G G G G G G G G G G G G G . G G G G G G G    
1026	. . . . . . . G G G G G G G G G G G G G G G G G G . G G G G G G G    
1027	. . . . . . . T T T T T T T T T T T T T T T T T T . T T T T T T T    
1028	. . . . . . . T T T T T T T T T T T T T T T T T T . T T T T T T T    
1029	. . . . . . . G G G G G G G G G G G G G G G G G G . G G G G G G G    
1030	. . . . . . . T T T T T T T T T T T T T T T T T T . T T T T T T T    
1031	. . . . . . . T T T T T T T T T T T T T T T T T T . T T T T T T T    
1032	. . . . . . . T T T T T T T T T T T T T T T T T T . T T T T T T T    
1033	. . . . . . . G G G G G G G G G G G G G G G G G G . G G G G G G G    
1034	. . . . . . . A A A A A A A A A A A A A A A A A A . A A A A A A A    
1035	. . . . . . . T T T T T T T T T T T T T T T T T T . T T T T T T T    
1036	. . . . . . . T T T T T T T T T T T T T T T T T T . T T T T T T T    
1037	. . . . . . . T T T T T T T T T T T T T T T T T T . T T T T T T T    
1038	. . . . . . . C C C C C C C C C C C C C C C C C C . C C C C C C C    
1039	. . . . . . . C C C C C C C C C C C C C C C C C C . C C C C C C C    
1040	. . . . . . . G G G G G G G G G G G G G G G G G G . G G G G G G G    
1041	. . . . . . . T T T T T T T T T T T T T T T T T T . T T T T T T T    
1042	. . . . . . . T T T T T T T T T T T T T T T T T T . T T T T T T T    
1043	. . . . . . . C C C C C C C C C C C C C C C C C C . C C C C C C C    
1044	. . . . . . . T T T T T T T T T T T T T T T T T T . T T T T T T T    
1045	. . . . . . . T T T T T T T T T T T T T T T T T T . T T T T T T T    
1046	. . . . . . . T T T T T T T T T T T T T T T T T T . T T T T T T T    
1047	. . . . . . . G G G G G G G G G G G G G G G G G G . G G G G G G G    
1048	. . . . . . . G G G G G G G G G G G G G G G G G G . G G G G G G G    
1049	. . . . . . . C C C C C C C C C C C C C C C C C C . C C C C C C C    
1050	. . . . . . . T T T T T T T T T T T T T T T T T T . T T T T T T T    
1051	. . . . . . . C C C C C C C C C C C C C C C C C C . C C C C C C C    
1052	. . . . . . . A A A A A A A A A A A A A A A A A A . A A A A A A A    
1053	. . . . . . . T T T T T T T T T T T T T T T T T T . T T T T T T T    
1054	. . . . . . . T T T T T T T T T T T T T T T T T T . T T T T T T T    
1055	. . . . . . . T T T T T T T T T T T T T T T T T T . T T T T T T T    
1056	. . . . . . . C C C C C C C C C C C C C C C C C C . C C C C C C C    
1057	. . . . . . . A A A A A A A A A A A A A A A A A A . A A A A A A A    
1058	. . . . . . . A A A A A A A A A A A A A A A A A A . A A A A A A A    
1059	. . . . . . . A A A A A A A A A A A A A A A A A A . A A A A A A A    
1060	. . . . . . . T T T T T T T T T T T T T T T T T T . T T T T T T T    
1061	. . . . . . . G G G G G G G G G G G G G G G G G G . G G G G G G G    
1062	. . . . . . . A A A A A A A A A A A A A A A A A A . A A A A A A A    
1063	. . . . . . . C C C C C C C C C C C C C C C C C C . C C C C C C C    
1064	. . . . . . . A A A A A A A A A A A A A A A A A A . A A A A A A A    
1065	. . . . . . . A A A A A A A A A A A A A A A A A A . A A A A A A A    
1066	. . . . . . . A A A A A A A A A A A A A A A A A A . A A A A A A A    
1067	. . . . . . . A A A A A A A A A A A A A A A A A A . A A A A A A A    
1068	. . . . . . . T T T T T T T T T T T T T T T T T T . T T T T T T T    
1069	. . . . . . . G G G G G G G G G G G G G G G G G G . G G G G G G G    
1070	. . . . . . . T T T T T T T T T T T T T T T T T T . T T T T T T T    
1071	. . . . . . . T T T T T T T T T T T T T T T T T T . T T T T T T T    
1072	. . . . . . . T T T T T T T T T T T T T T T T T T . T T T T T T T    
1073	. . . . . . . G G G G G G G G G G G G G G G G G G . G G G G G G G    
1074	. . . . . . . C C C C C C C C C C C C C C C C C C . C C C C C C C    
1075	. . . . . . . A A A A A A A A A A A A A A A A A A . A A A A A A A    
1076	. . . . . . . . A A A A A A A A A A A A A A A A A . A A A A A A A    
1077	. . . . . . . . A A A A A A A A A A A A A A A A A . A A A A A A A    
1078	. . . . . . . . A A A A A A A A A A A A A A A A A . A A A A A A A    
1079	. . . . . . . . G G G G G G G G G G G G G G G G G . G G G G G G G    
1080	. . . . . . . . A A A A A A A A A A A A A A A A A . A A A A A A A    
1081	. . . . . . . . A A A A A A A A A A A A A A A A A . A A A A A A A    
1082	. . . . . . . . A A A A A A A A A A A A A A A A A . A A A A A A A    
1083	. . . . . . . . T T T T T T T T T T T T T T T T T . T T T T T T T    
1084	. . . . . . . . C C C C C C C C C C C C C C C C C . C C C C C C C    
1085	. . . . . . . . A A A A A A A A A A A A A A A A A . A A A A A A A    
1086	. . . . . . . . T T T T T T T T T T T T T T T T T . T T T T T T T    
1087	. . . . . . . . G G G G G G G G G G G G G G G G G . G G G G G G G    
1088	. . . . . . . . G G G G G G G G G G G G G G G G G . G G G G G G G    
1089	. . . . . . . . T T T T T T T T T T T T T T T T T . T T T T T T T    
1090	. . . . . . . . A A A A A A A A A A A A A A A A A . A A A A A A A    
1091	. . . . . . . . A A A A A A A A A A A A A A A A A . A A A A A A A    
1092	. . . . . . . . T T T T T T T T T T T T T T T T T . T T T T T T T    
1093	. . . . . . . . T T T T T T T T T T T T T T T T T . T T T T T T T    
1094	. . . . . . . . A A A A A A A A A A A A A A A A A . A A A A A A A    
1095	. . . . . . . . T T T T T T T T T T T T T T T T T . T T T T T T T    
1096	. . . . . . . . T T T T T T T T T T T T T T T T T . T T T T T T T    
1097	. . . . . . . . G G G G G G G G G G G G G G G G G . G G G G G G G    
1098	. . . . . . . . G G G G G G G G G G G G G G G G G . G G G G G G G    
1099	. . . . . . . . C C G C G G G G G C C C C C G C G . G C G G G G G  10	 transversion  
1100	. . . . . . . . C C C C C C C C C C C C C C C C C . C C C C C C C    
1101	. . . . . . . . T T T T T T T T T T T T T T T T T . T T T T T T T    
1102	. . . . . . . . T T T T T T T T T T T T T T T T T . T T T T T T T    
1103	. . . . . . . . T T T T T T T T T T T T T T T T T . T T T T T T T    
1104	. . . . . . . . T T T T T T T T T T T T T T T T T . T T T T T T T    
1105	. . . . . . . . T T T T T T T T T T T T T T T T T . T T T T T T T    
1106	. . . . . . . . C C C C C C C C C C C C C C C C C . C C C C C C C    
1107	. . . . . . . . A A A A A A A A A A A A A A A A A . A A A A A A A    
1108	. . . . . . . . T T T T T T T T T T T T T T T T T . T T T T T T T    
1109	. . . . . . . . T T T T T T T T T T T T T T T T T . T T T T T T T    
1110	. . . . . . . . A A A A A A A A A A A A A A A A A . A A A A A A A    
1111	. . . . . . . . C C C C C C C C C C C C C C C C C . C C C C C C C    
1112	. . . . . . . . C C C C C C C C C C C C C C C C C . C C C C C C C    
1113	. . . . . . . . T T T T T T T T T T T T T T T T T . T T T T T T T    
1114	. . . . . . . . T T T T T T T T T T T T T T T T T . T T T T T T T    
1115	. . . . . . . . A A A A A A A A A A A A A A A A A . A A A A A A A    
1116	. . . . . . . . C C C C C C C C C C C C C C C C C . C C C C C C C    
1117	. . . . . . . . C C C C C C C C C C C C C C C C C . C C C C C C C    
1118	. . . . . . . . C C C C C C C C C C C C C C C C C . C C C C C C C    
1119	. . . . . . . . A A A A A A A A A A A A A A A A A . A A A A A A A    
1120	. . . . . . . . T T T T T T T T T T T T T T T T T . T T T T T T T    
1121	. . . . . . . . C C C C C C C C C C C C C C C C C . C C C C C G C    
1122	. . . . . . . . T T T T T T T T T T T T T T T T T . T T T T T T T    
1123	. . . . . . . . G G G G G G G G G G G G G G G G G . G G G G G G G    
1124	. . . . . . . . C C C C C C C C C C C C C C C C C . C C C C C C C    
1125	. . . . . . . . A A A A A A A A A A A A A A A A A . A A A A A A A    
1126	. . . . . . . . C C C C C C C C C C C C C C C C C . C C C C C C C    
1127	. . . . . . . . T T T T T T T T T T T T T T T T T . T T T T T T T    
1128	. . . . . . . . A A A A A A A A A A A A A A A A A . A A A A A A A    
1129	. . . . . . . . A A A A A A A A A A A A A A A A A . A A A A A A A    
1130	. . . . . . . . T T T T T T T T T T T T T T T T T . T T T T T T T    
1131	. . . . . . . . T T T T T T T T T T T T T T T T T . T T T T T T T    
1132	. . . . . . . . G G G G G G G G G G G G G G G G G . G G G G G G G    
1133	. . . . . . . . T T T T T T T T T T T T T T T T T . T T T T T T T    
1134	. . . . . . . . G G G G G G G G G G G G G G G G G . G G G G G G G    
1135	. . . . . . . . T T T T T T T T T T T T T T T T T . T T T T T T T    
1136	. . . . . . . . C C C C C C C C C C C C C C C C C . C C C C C C C    
1137	. . . . . . . . A A A A A A A A A A A A A A A A A . A A A A A A A    
1138	. . . . . . . . A A A A A A A A A A A A A A A A A . A A A A A A A    
1139	. . . . . . . . C C C C C C C C C C C C C C C C C . C C C C C C C    
1140	. . . . . . . . A A A A A A A A A A A A A A A A A . A A A A A A A    
1141	. . . . . . . . G G G G G G G G G G G G G G G G G . G G G G G G G    
1142	. . . . . . . . T T T T T T T T T T T T T T T T T . T T T T T T T    
1143	. . . . . . . . A A A A A A A A A A A A A A A A A . A A A A A A A    
1144	. . . . . . . . A A A A A A A A A A A A A A A A A . A A A A A A A    
1145	. . . . . . . . T T T T T T T T T T T T T T T T T . T T T T T T T    
1146	. . . . . . . . G G G G G G G G G G G G G G G G G . G G G G G G G    
1147	. . . . . . . . C C C C C C C C C C C C C C C C C . C C C C C C C    
1148	. . . . . . . . A A A A A A A A A A A A A A A A A . A A A A A A A    
1149	. . . . . . . . T T T T T T T T T T T T T T T T T . T T T T T T T    
1150	. . . . . . . . C C C C C C C C C C C C C C C C C . C C C C C C C    
1151	. . . . . . . . A A A A A A A A A A A A A A A A A . A A A A A A A    
1152	. . . . . . . . A A A A A A A A A A A A A A A A A . A A A A A A A    
1153	. . . . . . . . C C C C C C C C C C C C C C C C C . C C C C C C C    
1154	. . . . . . . . T T T T T T T T T T T T T T T T T . T T T T T T T    
1155	. . . . . . . . T T T T T T T T T T T T T T T T T . T T T T T T T    
1156	. . . . . . . . C C C C C C C C C C C C C C C C C . C C C C C C C    
1157	. . . . . . . . C C C C C C C C C C C C C C C C C . C C C C C C C    
1158	. . . . . . . . A A A A A A A A A A A A A A A A A . A A A A A A A    
1159	. . . . . . . . T T T T T T T T T T T T T T T T T . T T T T T T T    
1160	. . . . . . . . G G G G G G G G G G G G G G G G G . G G G G G G G    
1161	. . . . . . . . T T T T T T T T T T T T T T T T T . T T T T T T T    
1162	. . . . . . . . T T T T T T T T T T T T T T T T T . T T T T T T T    
1163	. . . . . . . . T T T T T T T T T T T T T T T T T . T T T T T T T    
1164	. . . . . . . . T T T T T T T T T T T T T T T T T . T T T T T T T    
1165	. . . . . . . . C C C C C C C C C C C C C C C C C . C C C C C C C    
1166	. . . . . . . . T T T T T T T T T T T T T T T T T . T T T T T T T    
1167	. . . . . . . . T T T T T T T T T T T T T T T T T . T T T T T T T    
1168	. . . . . . . . T T T T T T T T T T T T T T T T T . T T T T T T T    
1169	. . . . . . . . C C C C C C C C C C C C C C C C C . C C C C C C C    
1170	. . . . . . . . T T T T T T T T T T T T T T T T T . T T T T T T T    
1171	. . . . . . . . C C C C C C C C C C C C C C C C C . C C C C C C C    
1172	. . . . . . . . C C C C C C C C C C C C C C C C C . C C C C C C C    
1173	. . . . . . . . T T T T T T T T T T T T T T T T T . T T T T T T T    
1174	. . . . . . . . A A A A A A A A A A A A A A A A A . A A A A A A A    
1175	. . . . . . . . T T T T T T T T T T T T T T T T T . T T T T T T T    
1176	. . . . . . . . T T T T T T T T T T T T T T T T T . T T T T T T T    
1177	. . . . . . . . A A A A A A A A A A A A A A A A A . A A A A A A A    
1178	. . . . . . . . C C C C C C C C C C C C C C C C C . C C C C C C C    
1179	. . . . . . . . T T T T T T T T T T T T T T T T T . T T T T T T T    
1180	. . . . . . . . T T T T T T T T T T T T T T T T T . T T T T T T T    
1181	. . . . . . . . T T T T T T T T T T T T T T T T T . T T T T T T T    
1182	. . . . . . . . T T T T T T T T T T T T T T T T T . T T T T T T T    
1183	. . . . . . . . A A A A A A A A A A A A A A A A A . A A A A A A A    
1184	. . . . . . . . A A A A A A A A A A A A A A A A A . A A A A A A A    
1185	. . . . . . . . G G G G G G G G G G G G G G G G G . G G G G G G G    
1186	. . . . . . . . A A A A A A A A A A A A A A A A A . A A A A A A A    
1187	. . . . . . . . A A A A A A A A A A A A A A A A A . A A A A A A A    
1188	. . . . . . . . G G G G G G G G G G G G G G G G G . G G G G G G G    
1189	. . . . . . . . T T T T T T T T T T T T T T T T T . T T T T T T T    
1190	. . . . . . . . T T T T T T T T T T T T T T T T T . T T T T T T T    
1191	. . . . . . . . G G G G G G G G G G G G G G G G G . G G G G G G G    
1192	. . . . . . . . T T T T T T T T T T T T T T T T T . T T T T C T C    
1193	. . . . . . . . C C C C C C C C C C C C C C C C C . C C C C C C C    
1194	. . . . . . . . A A A A A A A A A A A A A A A A A . A A A A A A A    
1195	. . . . . . . . A A A A A A A A A A A A A A A A A . A A A A A A A    
1196	. . . . . . . . T A A A A A A A A A A A A A A A A . A A A A A A A    
1197	. . . . . . . . . T T T T T T T T T T T T T T T T . T T T T T T T    
1198	. . . . . . . . . A A A A A A A A A A A A A A A A . A A A A A A A    
1199	. . . . . . . . . A A A A A A A A A A A A A A A A . A A A A A A A    
1200	. . . . . . . . . - C - C C C C C - - - C C C - C . C - C C C C C  7	 indel  
1201	. . . . . . . . . - A - A A A A A - - - A A A - A . A - A A A A A  7	 indel  
1202	. . . . . . . . . T T T T T T T T T T T T T T T T . T T T T T T T    
1203	. . . . . . . . . A A A A A A A A A A A A A A A A . A A A A A A A    
1204	. . . . . . . . . T T T T T T T T T T T T T T T T . T T T T T T T    
1205	. . . . . . . . . T T T T T T T T T T T T T T T T . T T T T T T T    
1206	. . . . . . . . . T T T T T T T T T T T T T T T T . T T T T T T T    
1207	. . . . . . . . . A A A A A A A A A A A A A A A A . A A A A A A A    
1208	. . . . . . . . . C C C C C C C C C C C C C C C C C C C C C C C C    
1209	. . . . . . . . . T T T T T T T T T T T T T T T T T T T T T T T T    
1210	. . . . . . . . . . A A A A A A A A A A A A A A A A A A A A A A A    
1211	. . . . . . . . . . A A A A A A A A A A A A A A A A A A A A A A A    
1212	. . . . . . . . . . G G G G G G G G G G G G G G G G G G G G G G G    
1213	. . . . . . . . . . T T T T T T T T T T T T T T T T T T T T T T T    
1214	. . . . . . . . . . G G G G G G G G G G G G G G G G G G G G G G G    
1215	. . . . . . . . . . A A A A A A A A A A A A A A A A A A A A A A A    
1216	. . . . . . . . . . C C C C C C C C C C C C C C C C C C C C C C C    
1217	. . . . . . . . . . T T T T T T T T T T T T T T T T T T T T T T T    
1218	. . . . . . . . . . T T T T T T T T T T T T T T T T T T T T T T T    
1219	. . . . . . . . . . T T T T T T T T T T T T T T T T T T T T T T T    
1220	. . . . . . . . . . G G G G G G G G G G G G G G G G G G G G G G G    
1221	. . . . . . . . . . T T T T T T T T T T T T T T T T T T T T T T T    
1222	. . . . . . . . . . T T T T T T T T T T T T T T T T T T T T T T T    
1223	. . . . . . . . . . T T T T T T T T T T T T T T T T T T T T T T T    
1224	. . . . . . . . . . T T T T T T T T T T T T T T T T T T T T T T T    
1225	. . . . . . . . . . A A A A A A A A A A A A A A A A A A A A A A A    
1226	. . . . . . . . . . T T T T T T T T T T T T T T T T T T T T T T T    
1227	. . . . . . . . . . A A A A A A A A A A A A A A A A A A A A A A A    
1228	. . . . . . . . . . T T T T T T T T T T T T T T T T T T T T T T T    
1229	. . . . . . . . . . G G G G G G G G G G G G G G G G G G G G G G G    
1230	. . . . . . . . . . G G G G G G G G G G G G G G G G G G G G G G G    
1231	. . . . . . . . . . G G G G G G G G G G G G G G G G G G G G G G G    
1232	. . . . . . . . . . A A A A A A A A A A A A A A A A A A A A A A A    
1233	. . . . . . . . . . A A A A A A A A A A A A A A A A A A A A A A A    
1234	. . . . . . . . . . G G G G G G G G G G G G G G G G G G G G G G G    
1235	. . . . . . . . . . C C C C C C C C C C C C C C C C C C C C C C C    
1236	. . . . . . . . . . T T T T T T T T T T T T T T T T T T T T T T T    
1237	. . . . . . . . . . G G G G G G G G G G G G G G G G G G G G G G G    
1238	. . . . . . . . . . C C C C C C C C C C C C C C C C C C C C C C C    
1239	. . . . . . . . . . A A A A A A A A A A A A A A A A A A A A A A A    
1240	. . . . . . . . . . A A A A A A A A A A A A A A A A A A A A A A A    
1241	. . . . . . . . . . G G G G G G G G G G G G G G G G G G G G G G G    
1242	. . . . . . . . . . G G G G G G G G G G G G G G G G G G G G G G G    
1243	. . . . . . . . . . C C C C C C C C C C C C C C C C C C C C C C C    
1244	. . . . . . . . . . T T T T T T T T T T T T T T T T T T T T T T T    
1245	. . . . . . . . . . T T T T T T T T T T T T T T T T T T T T T T T    
1246	. . . . . . . . . . T T T T T T T T T T T T T T T T T T T T T T T    
1247	. . . . . . . . . . C C C C C C C C C C C C C C C C C C C C C C C    
1248	. . . . . . . . . . A A A A A A A A A A A A A A A A A A A A A A A    
1249	. . . . . . . . . . T T T T T T T T T T T T T T T T T T T T T T T    
1250	. . . . . . . . . . C C C C C C C C C C C C C C C C C C C C C C C    
1251	. . . . . . . . . . T T T T T T T T T T T T T T T T T T T T T T T    
1252	. . . . . . . . . . T T T T T T T T T T T T T T T T T T T T T T T    
1253	. . . . . . . . . . T T T T T T T T T T T T T T T T T T T T T T T    
1254	. . . . . . . . . . C C C C C C C C C C C C C C C C C C C C C C C    
1255	. . . . . . . . . . C C C C C C C C C C C C C C C C C C C C C C C    
1256	. . . . . . . . . . C C C C C C C C C C C C C C C C C C C C C C C    
1257	. . . . . . . . . . A A A A A A A A A A A A A A A A A A A A A A A    
1258	. . . . . . . . . . T T T T T T T T T T T T T T T T T T T T T T T    
1259	. . . . . . . . . . A A A A A A A A A A A A A A A A A A A A A A A    
1260	. . . . . . . . . . A A A A A A A A A A A A A A A A A A A A A A A    
1261	. . . . . . . . . . T T T T T T T T T T T T T T T T T T T T T T T    
1262	. . . . . . . . . . A A A A A A A A A A A A A A A A A A A A A A A    
1263	. . . . . . . . . . T T T T T T T T T T T T T T T T T T T T T T T    
1264	. . . . . . . . . . A A A A A A A A A A A A A A A A A A A A A A A    
1265	. . . . . . . . . . T T T T T T T T T T T T T T T T T T T T T T T    
1266	. . . . . . . . . . T T T T T T T T T T T T T T T T T T T T T T T    
1267	. . . . . . . . . . C C T C C C C C C C C C C C C C C C C C C A C    
1268	. . . . . . . . . . T T T T T T T T T T T T T T T T T T T T T T T    
1269	. . . . . . . . . . A A A A A A A A A A A A A A A A A A A A A A A    
1270	. . . . . . . . . . A A A A A A A A A A A A A A A A A A A A A A A    
1271	. . . . . . . . . . T T T T T T T T T T T T T T T T T T T T T T T    
1272	. . . . . . . . . . T T T T T T T T T T T T T T T T T T T T T T T    
1273	. . . . . . . . . . C C C C C C C C C C C C C C C C C C C C C C C    
1274	. . . . . . . . . . T T T T T T T T T T T T T T T T T T T T T T T    
1275	. . . . . . . . . . T T T T T T T T T T T T T T T T T T T T T T T    
1276	. . . . . . . . . . G G G G G G G G G G G G G G G G G G G G G G G    
1277	. . . . . . . . . . T T T T T T T T T T T T T T T T T T T T T T T    
1278	. . . . . . . . . . T T T T T T T T T T T T T T T T T T T T T T T    
1279	. . . . . . . . . . T T T T T T T T T T T T T T T T T T T T T T T    
1280	. . . . . . . . . . T T T T T T T T T T T T T T T T T T T T T T T    
1281	. . . . . . . . . . G G G G G G G G G G G G G G G G G G G G G G G    
1282	. . . . . . . . . . T T T T T T T T T T T T T T T T T T T T T T T    
1283	. . . . . . . . . . T T T T T T T T T T T T T T T T T T T T T T T    
1284	. . . . . . . . . . T T T T T T T T T T T T T T T T T T T T T T T    
1285	. . . . . . . . . . T T T T T T T T T T T T T T T T T T T T T T T    
1286	. . . . . . . . . . T T T T T T T T T T T T T T T T T T T T T T T    
1287	. . . . . . . . . . T T T T T T T T T T T T T T T T T T T T T T T    
1288	. . . . . . . . . . T T T T T T T T T T T T T T T T T T T T T T T    
1289	. . . . . . . . . . G G G G G G G G G G G G G G G G G G G G G G G    
1290	. . . . . . . . . . A A A A A A A A A A A A A A A A A A A A A A A    
1291	. . . . . . . . . . A A A A A A A A A A A A A A A A A A A A A A A    
1292	. . . . . . . . . . T T T T T T T T T T T T T T T T T T T T T T T    
1293	. . . . . . . . . . A A A A A A A A A A A A A A A A A A A A A A A    
1294	. . . . . . . . . . A A A A A A A A A A A A A A A A A A A A A A A    
1295	. . . . . . . . . . T T T T T T T T T T T T T T T T T T T T T T T    
1296	. . . . . . . . . . G G G G G G G G G G G G G G G G G G G G G G G    
1297	. . . . . . . . . . A A A A A A A A A A A A A A A A A A A A A A A    
1298	. . . . . . . . . . A A A A A A A A A A A A A A A A A A A A A A A    
1299	. . . . . . . . . . G G G G G G G G G G G G G G G G G G G G G G G    
1300	. . . . . . . . . . C C C C C C C C C C C C C C C C C C C C C C C    
1301	. . . . . . . . . . T T T T T T T T T T T T T T T T T T T T T T T    
1302	. . . . . . . . . . G G G G G G G G G G G G G G G G G G G G G G G    
1303	. . . . . . . . . . T T T T T T T T T T T T T T T T T T T T T T T    
1304	. . . . . . . . . . G G G G G G G G G G G G G G G G G G G G G G G    
1305	. . . . . . . . . . G G G G G G G G G G G G G G G G G G G G G G G    
1306	. . . . . . . . . . A A A A A A A A A A A A A A A A A A A A A A A    
1307	. . . . . . . . . . A A A A A A A A A A A A A A A A A A A A A A A    
1308	. . . . . . . . . . A A A A A A A A A A A A A A A A A A A A A A A    
1309	. . . . . . . . . . A A A A A A A A A A A A A A A A A A A A A A A    
1310	. . . . . . . . . . C T T T T T T T T T T T T T T T T T T T T T T    
1311	. . . . . . . . . . . G G G G G G G G G G G G G G G G G G G G G G    
1312	. . . . . . . . . . . T T T T T T T T T T T T T T T T T T T T T T    
1313	. . . . . . . . . . . G G G G G G G G G G G G G G G G G G G G G G    
1314	. . . . . . . . . . . T T T T T T T T T T T T T T T T T T T T T T    
1315	. . . . . . . . . . . G G G G G G G G G G G G G G G G G G G G G G    
1316	. . . . . . . . . . . T T T T T T T T T T T T T T T T T T T T T T    
1317	. . . . . . . . . . . T T T T T T T T T T T T T T T T T T T T T T    
1318	. . . . . . . . . . . T T T T T T T T T T T T T T T T T T T T T T    
1319	. . . . . . . . . . . A A A A A A A A A A A A A A A A A A A A A A    
1320	. . . . . . . . . . . T T T T C C T T T T T T T T T T T T T T T T    
1321	. . . . . . . . . . . A A A A A A A A A A A A A A A A A A A A A A    
1322	. . . . . . . . . . . G G G G G G G G G G G G G G G G G G G G G G    
1323	. . . . . . . . . . . A A A A A A A A A A A A A A A A A A A A A A    
1324	. . . . . . . . . . . T A A A A A T A A A A A A A A A A A A A A A    
1325	. . . . . . . . . . . T T T T T T T T T T T T T T T T T T T T T T    
1326	. . . . . . . . . . . T T T T T T T T T T T T T T T T T T T T T T    
1327	. . . . . . . . . . . T T T T T T T T T T T T T T T T T T T T T T    
1328	. . . . . . . . . . . G G G G G G G G G G G G G G G G G G G G G G    
1329	. . . . . . . . . . . G G G G G G G G G G G G G G G G G G G G G G    
1330	. . . . . . . . . . . G G G G G G G G G G G G G G G G G G G G G G    
1331	. . . . . . . . . . . A A A A A A A A A A A A A A A A A A A A A A    
1332	. . . . . . . . . . . A A A A A A A A A A A A A A A A A A A A A A    
1333	. . . . . . . . . . . A A A A A A A A A A A A A A A A A A A A A A    
1334	. . . . . . . . . . . . T T T T T T T T T T T T T T T T T T T T T    
1335	. . . . . . . . . . . . G G G G G G G G G G G G G G G G G G G G G    
1336	. . . . . . . . . . . . A A A A A A A A A A A A A A A A A A A A A    
1337	. . . . . . . . . . . . G G G G G G G G G G G G G G G G G G G G G    
1338	. . . . . . . . . . . . G G G G G G G G G G G G G G G G G G G G G    
1339	. . . . . . . . . . . . A A A A A A A A A A A A A A A A A A A A A    
1340	. . . . . . . . . . . . G G G G G G G G G G G G G G G G G G G G G    
1341	. . . . . . . . . . . . A A A A A A A A A A A A A A A A A A A A A    
1342	. . . . . . . . . . . . C C C C C C C C C C C C C C C C C C C C C    
1343	. . . . . . . . . . . . T T T T T T T T T T T T T T T T T T T T T    
1344	. . . . . . . . . . . . T T T T T T T T T T T T T T T T T T T T T    
1345	. . . . . . . . . . . . G G G G G G G G G G G G G G G G G G G G G    
1346	. . . . . . . . . . . . A A A A A A A A A A A A A A A A A A A A A    
1347	. . . . . . . . . . . . T T T T T T T T T T T T T T T T T T T T T    
1348	. . . . . . . . . . . . T T T T T T T T T T T T T T T T T T T T T    
1349	. . . . . . . . . . . . T T T T T T T T T T T T T T T T T T T T T    
1350	. . . . . . . . . . . . C C C C C C C C C C C C C C C C C C C C C    
1351	. . . . . . . . . . . . T T T T T T T T T T T T T T T T T T T T T    
1352	. . . . . . . . . . . . G G G G G G G G G G G G G G G G G G G G G    
1353	. . . . . . . . . . . . T T T T T T T T T T T T T T T T T T T T T    
1354	. . . . . . . . . . . . C C C C C C C C C C C C C C C C C C C C C    
1355	. . . . . . . . . . . . T T T T T T T T T T T T T T T T T T T T T    
1356	. . . . . . . . . . . . T T T T T T T T T T T T T T T T T T T T T    
1357	. . . . . . . . . . . . G G G G G G G G G G G G G G G G G G G G G    
1358	. . . . . . . . . . . . A A A A A A A A A A A A A A A A A A A A A    
1359	. . . . . . . . . . . . G G G G G G G G G G G G G G G G G G G G G    
1360	. . . . . . . . . . . . G G G G G G G G G G G G G G G G G G G G G    
1361	. . . . . . . . . . . . T T T T T T T T T T T T T T T T T T T T T    
1362	. . . . . . . . . . . . T T T T T T T T T T T T T T T T T T T T T    
1363	. . . . . . . . . . . . G G G G G G G G G G G G G G G G G G G G G    
1364	. . . . . . . . . . . . A A A A A A A A A A A A A A A A A A A A A    
1365	. . . . . . . . . . . . A A A A A A A A A A A A A A A A A A A A A    
1366	. . . . . . . . . . . . A A A A A A A A A A A A A A T A A A A A A    
1367	. . . . . . . . . . . . A A A A A A A A A A A A A A A A A A A A A    
1368	. . . . . . . . . . . . T T T T T T T T T T T T T T T T T T T T T    
1369	. . . . . . . . . . . . A A A A A A A A A A A A A A A A A A A A A    
1370	. . . . . . . . . . . . T T T T T T T T T T T T T T T T T T T T T    
1371	. . . . . . . . . . . . G G G G G G G G G G G G G G G G G G G G G    
1372	. . . . . . . . . . . . T T T T T T T T T T T T T T T T T T T T T    
1373	. . . . . . . . . . . . T T T T T T T T T T T T T T T T T T T T T    
1374	. . . . . . . . . . . . G G G G G G G G G G G G G G G G G G G G G    
1375	. . . . . . . . . . . . T T T T T T T T T T T T T T T T T T T T T    
1376	. . . . . . . . . . . . A A A A A A A A A A A A A A A A A A A A A    
1377	. . . . . . . . . . . . C C C C C C C C C C C C C C C C C C C C C    
1378	. . . . . . . . . . . . T T T T T T T T T T T T T T T T T T T T T    
1379	. . . . . . . . . . . . A A A A A A A A A A A A A A A A A A A A A    
1380	. . . . . . . . . . . . A A A A A A A A A A A A A A A A A A A A A    
1381	. . . . . . . . . . . . A A A A A A A A A A A A A A A A A A A A A    
1382	. . . . . . . . . . . . C C C C C C C C C C C C C C C C C C C C C    
1383	. . . . . . . . . . . . C C C C C C C C C C C C C C C C C C C C C    
1384	. . . . . . . . . . . . A A A A A A A A A A A A A A A A A A A A A    
1385	. . . . . . . . . . . . A A A A A A A A A A A A A A A A A A A A A    
1386	. . . . . . . . . . . . C C C C C C C C C C C C C C C C C C C C C    
1387	. . . . . . . . . . . . T T T T T C T T T T T T T T T T T T T T T    
1388	. . . . . . . . . . . . C C C C C C C C C C C C C C C C C C C C C    
1389	. . . . . . . . . . . . T T T T T T T T T T T T T T T T T T T T T    
1390	. . . . . . . . . . . . G G G G G G G G G G G G G G G G G G G G G    
1391	. . . . . . . . . . . . T T T T T T T T T T T T T T T T T T T T T    
1392	. . . . . . . . . . . . T T T T T T T T T T T T T T T T T T T T T    
1393	. . . . . . . . . . . . G G G G G G G G G G G G G G G G G G G G G    
1394	. . . . . . . . . . . . T T T T T T T T T T T T T T T T T T T T T    
1395	. . . . . . . . . . . . G G G G G G G G G G G G G G G G G G G G G    
1396	. . . . . . . . . . . . T T T T T T T T T T T T T T T T T T T T T    
1397	. . . . . . . . . . . . C C C C C C C C C C C C C C C C C C C C C    
1398	. . . . . . . . . . . . T T T T T T T T T T T T T T T T T T T T T    
1399	. . . . . . . . . . . . T T T T T T T T T T T T T T T T T T T T T    
1400	. . . . . . . . . . . . G G G G G G G G G G G G G G G G G G G G G    
1401	. . . . . . . . . . . . T T T T T T T T T T T T T T T T T T T T T    
1402	. . . . . . . . . . . . T T T T T T T T T T T T T T T T T T T T T    
1403	. . . . . . . . . . . . A A A A A A A A A A A A A A A A A A A A A    
1404	. . . . . . . . . . . . C C C C C C C C C C C C C C C C C C C C C    
1405	. . . . . . . . . . . . C C C C C C C C C C C C C C C C C C C C C    
1406	. . . . . . . . . . . . T T T T T T T T T T T T T T T T T T T T T    
1407	. . . . . . . . . . . . T T T T T T T T T T T T T T T T T T T T T    
1408	. . . . . . . . . . . . G G G G G G G G G G G G G G G G G G G G G    
1409	. . . . . . . . . . . . T T T T T T T T T T T T T T T T T T T T T    
1410	. . . . . . . . . . . . C C C C C C C C C C C C C C C C C C C C C    
1411	. . . . . . . . . . . . G G G G G G G G G G G G G G G G G G G G G    
1412	. . . . . . . . . . . . C C C C C C C C C C C C C C C C C C C C C    
1413	. . . . . . . . . . . . A A A A A A A A A A A A A A A A A A A A A    
1414	. . . . . . . . . . . . A A A A A A A A A A A A A A A A A A A A A    
1415	. . . . . . . . . . . . A A T A A A A A A A A A A A A A A A A A A    
1416	. . . . . . . . . . . . T T . T T T T T T T T T T T T T T T T T T    
1417	. . . . . . . . . . . . G A . G G G G G G G G G G G G G G G G G G    
1418	. . . . . . . . . . . . T . . A A A A A A A A A A A A A A A A A A    
1419	. . . . . . . . . . . . . . . T T T T T T T T T T T T T T T T T T    
1420	. . . . . . . . . . . . . . . T T T T T T T T T T T T T T T T T T    
1421	. . . . . . . . . . . . . . . T T T T T T T T T T T T T T T T T T    
1422	. . . . . . . . . . . . . . . T T T T T T T T T T T T T T T T T T    
1423	. . . . . . . . . . . . . . . C C C C C C C C C C C C C C C C C C    
1424	. . . . . . . . . . . . . . . C C C C C C C C C C C C C C C C C C    
1425	. . . . . . . . . . . . . . . A A A A A A A A A A A A A A A A A A    
1426	. . . . . . . . . . . . . . . G G G G G G G G G G G G G G G G G G    
1427	. . . . . . . . . . . . . . . C C C C C C C C C C C C C C C C C C    
1428	. . . . . . . . . . . . . . . T T T T T T T T T T T T T T T T T T    
1429	. . . . . . . . . . . . . . . T T T T T T T T T T T T T T T T T T    
1430	. . . . . . . . . . . . . . . T T T T T T T T T T T T T T T T T T    
1431	. . . . . . . . . . . . . . . T T T T T T T T T T T T T T T T T T    
1432	. . . . . . . . . . . . . . . A A A A A A A A A A A A A A A A A A    
1433	. . . . . . . . . . . . . . . T T T T T T T T T T T T T T T T T T    
1434	. . . . . . . . . . . . . . . C C C C C C C C C C C C C C C C C C    
1435	. . . . . . . . . . . . . . . A A A A A A A A A A A A A A A A A A    
1436	. . . . . . . . . . . . . . . A A A A A A A A A A A A A A A A A A    
1437	. . . . . . . . . . . . . . . T T T T T T T T T T T T T T T T T T    
1438	. . . . . . . . . . . . . . . G G G G G G G G G G G G G G G G G G    
1439	. . . . . . . . . . . . . . . A A A A A A A A A A A A A A A A A A    
1440	. . . . . . . . . . . . . . . T T T T T T T T T T T T T T T T T T    
1441	. . . . . . . . . . . . . . . A A A A A A A A A A A A A A A A A A    
1442	. . . . . . . . . . . . . . . A A A A A A A A A A A A A A A A A A    
1443	. . . . . . . . . . . . . . . A A A A A A A A A A A A A A A A A A    
1444	. . . . . . . . . . . . . . . A A A A A A A A A A A A A A A A A A    
1445	. . . . . . . . . . . . . . . C C C C C C C C C C C C C C C C C C    
1446	. . . . . . . . . . . . . . . A A A A A A A A A A A A A A A A A A    
1447	. . . . . . . . . . . . . . . A A A A A A A A A A A A A A A A A A    
1448	. . . . . . . . . . . . . . . T T T T T T T T T T T T T T T T T T    
1449	. . . . . . . . . . . . . . . T T T T T T T T T T T T T T T T T T    
1450	. . . . . . . . . . . . . . . G G G G G G G G G G G G G G G G G G    
1451	. . . . . . . . . . . . . . . T T T T T T T T T T T T T T T T T T    
1452	. . . . . . . . . . . . . . . A A A A A A A A A A A A A A A A A A    
1453	. . . . . . . . . . . . . . . . C C C C C C C C C C C C C C C C C    
1454	. . . . . . . . . . . . . . . . A A A A A A A A A A A A A A A A A    
1455	. . . . . . . . . . . . . . . . T T T T T T T T T T T T T T T T T    
1456	. . . . . . . . . . . . . . . . T T T T T T T T T T T T T T T T T    
1457	. . . . . . . . . . . . . . . . T T T T T T T T T T T T T T T T T    
1458	. . . . . . . . . . . . . . . . C T T T T T T T T T T T T T T T T    
1459	. . . . . . . . . . . . . . . . . C C C C C C C C C C C C C C C C    
1460	. . . . . . . . . . . . . . . . . . A . A A A A A A A A . A A A A    
1461	. . . . . . . . . . . . . . . . . . T . T T T T T T . T . T T T T    
1462	. . . . . . . . . . . . . . . . . . T . T T T T T T . T . T T T T    
1463	. . . . . . . . . . . . . . . . . . . . . . . . . . . . . . A A A    
1464	. . . . . . . . . . . . . . . . . . . . . . . . . . . . . . T T T    
1465	. . . . . . . . . . . . . . . . . . . . . . . . . . . . . . C C C    
1466	. . . . . . . . . . . . . . . . . . . . . . . . . . . . . . T T T    
1467	. . . . . . . . . . . . . . . . . . . . . . . . . . . . . . T T T    
1468	. . . . . . . . . . . . . . . . . . . . . . . . . . . . . . T T T    
1469	. . . . . . . . . . . . . . . . . . . . . . . . . . . . . . T T T    
1470	. . . . . . . . . . . . . . . . . . . . . . . . . . . . . . G G G    
1471	. . . . . . . . . . . . . . . . . . . . . . . . . . . . . . T T T    
1472	. . . . . . . . . . . . . . . . . . . . . . . . . . . . . . C C C    
1473	. . . . . . . . . . . . . . . . . . . . . . . . . . . . . . A A A    
1474	. . . . . . . . . . . . . . . . . . . . . . . . . . . . . . A A A    
1475	. . . . . . . . . . . . . . . . . . . . . . . . . . . . . . T T T    
1476	. . . . . . . . . . . . . . . . . . . . . . . . . . . . . . G G G    
1477	. . . . . . . . . . . . . . . . . . . . . . . . . . . . . . G G G    
1478	. . . . . . . . . . . . . . . . . . . . . . . . . . . . . . A A A    
1479	. . . . . . . . . . . . . . . . . . . . . . . . . . . . . . T T T    
1480	. . . . . . . . . . . . . . . . . . . . . . . . . . . . . . T T T    
1481	. . . . . . . . . . . . . . . . . . . . . . . . . . . . . . T T T    
1482	. . . . . . . . . . . . . . . . . . . . . . . . . . . . . . T T T    
1483	. . . . . . . . . . . . . . . . . . . . . . . . . . . . . . A A A    
1484	. . . . . . . . . . . . . . . . . . . . . . . . . . . . . . T T T    
1485	. . . . . . . . . . . . . . . . . . . . . . . . . . . . . . T T T    
1486	. . . . . . . . . . . . . . . . . . . . . . . . . . . . . . T T T    
1487	. . . . . . . . . . . . . . . . . . . . . . . . . . . . . . T T T    
1488	. . . . . . . . . . . . . . . . . . . . . . . . . . . . . . C C C    
1489	. . . . . . . . . . . . . . . . . . . . . . . . . . . . . . T T T    
1490	. . . . . . . . . . . . . . . . . . . . . . . . . . . . . . G G G    
1491	. . . . . . . . . . . . . . . . . . . . . . . . . . . . . . T T T    
1492	. . . . . . . . . . . . . . . . . . . . . . . . . . . . . . A A A    
1493	. . . . . . . . . . . . . . . . . . . . . . . . . . . . . . T T T    
1494	. . . . . . . . . . . . . . . . . . . . . . . . . . . . . . A A A    
1495	. . . . . . . . . . . . . . . . . . . . . . . . . . . . . . C C C    
1496	. . . . . . . . . . . . . . . . . . . . . . . . . . . . . . A A A    
1497	. . . . . . . . . . . . . . . . . . . . . . . . . . . . . . T T T    
1498	. . . . . . . . . . . . . . . . . . . . . . . . . . . . . . G G G    
1499	. . . . . . . . . . . . . . . . . . . . . . . . . . . . . . A A A    
1500	. . . . . . . . . . . . . . . . . . . . . . . . . . . . . . T T T    
1501	. . . . . . . . . . . . . . . . . . . . . . . . . . . . . . G G G    
1502	. . . . . . . . . . . . . . . . . . . . . . . . . . . . . . A A A    
1503	. . . . . . . . . . . . . . . . . . . . . . . . . . . . . . T T T    
1504	. . . . . . . . . . . . . . . . . . . . . . . . . . . . . . A A A    
1505	. . . . . . . . . . . . . . . . . . . . . . . . . . . . . . A A A    
1506	. . . . . . . . . . . . . . . . . . . . . . . . . . . . . . T T T    
1507	. . . . . . . . . . . . . . . . . . . . . . . . . . . . . . C C C    
1508	. . . . . . . . . . . . . . . . . . . . . . . . . . . . . . A A A    
1509	. . . . . . . . . . . . . . . . . . . . . . . . . . . . . . A A A    
1510	. . . . . . . . . . . . . . . . . . . . . . . . . . . . . . T T T    
1511	. . . . . . . . . . . . . . . . . . . . . . . . . . . . . . A A A    
1512	. . . . . . . . . . . . . . . . . . . . . . . . . . . . . . G G G    
1513	. . . . . . . . . . . . . . . . . . . . . . . . . . . . . . T T T    
1514	. . . . . . . . . . . . . . . . . . . . . . . . . . . . . . T T T    
1515	. . . . . . . . . . . . . . . . . . . . . . . . . . . . . . G G G    
1516	. . . . . . . . . . . . . . . . . . . . . . . . . . . . . . T T T    
1517	. . . . . . . . . . . . . . . . . . . . . . . . . . . . . . G G G    
1518	. . . . . . . . . . . . . . . . . . . . . . . . . . . . . . A A A    
1519	. . . . . . . . . . . . . . . . . . . . . . . . . . . . . . A A A    
1520	. . . . . . . . . . . . . . . . . . . . . . . . . . . . . . . G G    
1521	. . . . . . . . . . . . . . . . . . . . . . . . . . . . . . . A A    
1522	. . . . . . . . . . . . . . . . . . . . . . . . . . . . . . . A A    
1523	. . . . . . . . . . . . . . . . . . . . . . . . . . . . . . . G T    
1524	. . . . . . . . . . . . . . . . . . . . . . . . . . . . . . . . G    
1525	. . . . . . . . . . . . . . . . . . . . . . . . . . . . . . . . T    
1526	. . . . . . . . . . . . . . . . . . . . . . . . . . . . . . . . T    
1527	. . . . . . . . . . . . . . . . . . . . . . . . . . . . . . . . T    
1528	. . . . . . . . . . . . . . . . . . . . . . . . . . . . . . . . G    
1529	. . . . . . . . . . . . . . . . . . . . . . . . . . . . . . . . C    
1530	. . . . . . . . . . . . . . . . . . . . . . . . . . . . . . . . C    
1531	. . . . . . . . . . . . . . . . . . . . . . . . . . . . . . . . T    
1532	. . . . . . . . . . . . . . . . . . . . . . . . . . . . . . . . G    
1533	. . . . . . . . . . . . . . . . . . . . . . . . . . . . . . . . T    
1534	. . . . . . . . . . . . . . . . . . . . . . . . . . . . . . . . T    
1535	. . . . . . . . . . . . . . . . . . . . . . . . . . . . . . . . G    
1536	. . . . . . . . . . . . . . . . . . . . . . . . . . . . . . . . C    
1537	. . . . . . . . . . . . . . . . . . . . . . . . . . . . . . . . A    
1538	. . . . . . . . . . . . . . . . . . . . . . . . . . . . . . . . A    
1539	. . . . . . . . . . . . . . . . . . . . . . . . . . . . . . . . A    
1540	. . . . . . . . . . . . . . . . . . . . . . . . . . . . . . . . T    
1541	. . . . . . . . . . . . . . . . . . . . . . . . . . . . . . . . C    
1542	. . . . . . . . . . . . . . . . . . . . . . . . . . . . . . . . T    
1543	. . . . . . . . . . . . . . . . . . . . . . . . . . . . . . . . C    
1544	. . . . . . . . . . . . . . . . . . . . . . . . . . . . . . . . T    
1545	. . . . . . . . . . . . . . . . . . . . . . . . . . . . . . . . A    
1546	. . . . . . . . . . . . . . . . . . . . . . . . . . . . . . . . G    
1547	. . . . . . . . . . . . . . . . . . . . . . . . . . . . . . . . T    
1548	. . . . . . . . . . . . . . . . . . . . . . . . . . . . . . . . G    
1549	. . . . . . . . . . . . . . . . . . . . . . . . . . . . . . . . T    
1550	. . . . . . . . . . . . . . . . . . . . . . . . . . . . . . . . T    
1551	. . . . . . . . . . . . . . . . . . . . . . . . . . . . . . . . T    
1552	. . . . . . . . . . . . . . . . . . . . . . . . . . . . . . . . T    
1553	. . . . . . . . . . . . . . . . . . . . . . . . . . . . . . . . G    
1554	. . . . . . . . . . . . . . . . . . . . . . . . . . . . . . . . C    
1555	. . . . . . . . . . . . . . . . . . . . . . . . . . . . . . . . G    
1556	. . . . . . . . . . . . . . . . . . . . . . . . . . . . . . . . G    
1557	. . . . . . . . . . . . . . . . . . . . . . . . . . . . . . . . G    
1558	. . . . . . . . . . . . . . . . . . . . . . . . . . . . . . . . A    
1559	. . . . . . . . . . . . . . . . . . . . . . . . . . . . . . . . A    
1560	. . . . . . . . . . . . . . . . . . . . . . . . . . . . . . . . C    
1561	. . . . . . . . . . . . . . . . . . . . . . . . . . . . . . . . A    
1562	. . . . . . . . . . . . . . . . . . . . . . . . . . . . . . . . T    
1563	. . . . . . . . . . . . . . . . . . . . . . . . . . . . . . . . T    
1564	. . . . . . . . . . . . . . . . . . . . . . . . . . . . . . . . A    
1565	. . . . . . . . . . . . . . . . . . . . . . . . . . . . . . . . A    
1566	. . . . . . . . . . . . . . . . . . . . . . . . . . . . . . . . A    
1567	. . . . . . . . . . . . . . . . . . . . . . . . . . . . . . . . T    
1568	. . . . . . . . . . . . . . . . . . . . . . . . . . . . . . . . A    
1569	. . . . . . . . . . . . . . . . . . . . . . . . . . . . . . . . A    
1570	. . . . . . . . . . . . . . . . . . . . . . . . . . . . . . . . T    
1571	. . . . . . . . . . . . . . . . . . . . . . . . . . . . . . . . T    
1572	. . . . . . . . . . . . . . . . . . . . . . . . . . . . . . . . G    
1573	. . . . . . . . . . . . . . . . . . . . . . . . . . . . . . . . T    
1574	. . . . . . . . . . . . . . . . . . . . . . . . . . . . . . . . T    
1575	. . . . . . . . . . . . . . . . . . . . . . . . . . . . . . . . G    
1576	. . . . . . . . . . . . . . . . . . . . . . . . . . . . . . . . G    
1577	. . . . . . . . . . . . . . . . . . . . . . . . . . . . . . . . C    
1578	. . . . . . . . . . . . . . . . . . . . . . . . . . . . . . . . A    
1579	. . . . . . . . . . . . . . . . . . . . . . . . . . . . . . . . T    
1580	. . . . . . . . . . . . . . . . . . . . . . . . . . . . . . . . A    
1581	. . . . . . . . . . . . . . . . . . . . . . . . . . . . . . . . G
```
